# Anti-vascular endothelial growth factor monotherapy or combined with verteporfin photodynamic therapy for retinal angiomatous proliferation: a systematic review with meta-analysis

**DOI:** 10.3389/fphar.2023.1141077

**Published:** 2023-06-12

**Authors:** Matteo Fallico, Iacopo Macchi, Andrea Maugeri, Giuliana Favara, Martina Barchitta, Roberta Magnano San Lio, Antonella Agodi, Andrea Russo, Antonio Longo, Teresio Avitabile, Niccolò Castellino, Michele Reibaldi, Francesco Pignatelli, Maria Vadalà, Clara Patanè, Marcella Nebbioso, Vincenza Bonfiglio

**Affiliations:** ^1^ Department of Ophthalmology, University of Catania, Catania, Italy; ^2^ Newcastle Eye Unit, Royal Victoria Infirmary, Newcastle Upon Tyne, United Kingdom; ^3^ Department of Medical and Surgical Sciences and Advanced Technologies “GF Ingrassia”, University of Catania, Catania, Italy; ^4^ Department of Surgical Sciences, Eye Clinic Section, University of Turin, Turin, Italy; ^5^ Eye Clinic, Hospital “SS Annunziata”, ASL Taranto, Taranto, Italy; ^6^ Department of Experimental Biomedicine and Clinical Neuroscience, Ophthalmology Section, University of Palermo, Palermo, Italy; ^7^ Department of Sense Organs, Faculty of Medicine and Dentistry, Sapienza University of Rome, Rome, Italy

**Keywords:** retinal angiomatous proliferation (RAP), anti vascular endothelial growth factor, verteporfin photodynamic therapy (V-PDT), monotherapy, combined therapy

## Abstract

**Purpose:** To assess functional and anatomical outcomes of intravitreal anti-Vascular Endothelial Growth Factor (anti-VEGF) monotherapy *versus* combined with verteporfin Photodynamic Therapy (PDT) for Retinal Angiomatous Proliferation (RAP).

**Methods:** Studies reporting outcomes of intravitreal anti-VEGF monotherapy and/or in combination with verteporfin PDT in RAP eyes with a follow-up ≥ 12 months were searched. The primary outcome was the mean change in best corrected visual acuity (BCVA) at 12 months. Mean change in central macular thickness (CMT) and mean number of injections were considered as secondary outcomes. The mean difference (MD) between pre- and post-treatment values was calculated along with 95% Confidence Interval (95% CI). Meta-regressions were performed to assess the influence of anti-VEGF number of injections on BCVA and CMT outcomes.

**Results:** Thirty-four studies were included. A mean gain of 5.16 letters (95% CI = 3.30–7.01) and 10.38 letters (95% CI = 8.02–12.75) was shown in the anti-VEGF group and combined group, respectively (anti-VEGF group vs. combined group, *p* < 0.01). A mean CMT reduction of 132.45 µm (95% CI = from −154.99 to −109.90) and 213.93 µm (95% CI = from −280.04 to −147.83) was shown in the anti-VEGF group and combined group, respectively (anti-VEGF group vs. combined group, *p* < 0.02). A mean of 4.9 injections (95% CI = 4.2–5.6) and 2.8 injections (95% CI = 1.3–4.4) were administered over a 12-month period in the anti-VEGF group and combined group, respectively. Meta-regression analyses showed no influence of injection number on visual and CMT outcomes. High heterogeneity was found across studies for both functional and anatomical outcomes.

**Conclusion:** A combined approach with anti-VEGF and PDT could provide better functional and anatomical outcomes in RAP eyes compared with anti-VEGF monotherapy.

## 1 Introduction

Retinal angiomatous proliferation (RAP) was firstly described by Yannuzzi et al. as a distinct form of neovascularage-related macular degeneration (nAMD). (Yannuzzi et al., 2001).

According to the anatomic classification, RAP is defined as “type 3 neovascularization”. (Freund et al., 2008). The peculiar characteristic of RAP is that it consists of two different neovascular foci, one originating in the deep retina and the other within the choroid. (Yannuzzi et al., 2008). Usually, the neovascular network originates in the deep retina and extends to choroidal neovessels through vascular anastomosis. (Freund et al., 2008). Natural course of RAP is different compared with other forms of nAMD, featuring a rapid progression to advanced stages and poor visual outcomes, especially in cases of inadequate treatment or delayed diagnosis. (Viola et al., 2009).

Intravitreal anti-vascular endothelial growth factor (anti-VEGF) therapy has become the first line treatment for nAMDand for RAP lesions as well. (Tsai et al., 2017; Reibaldi et al., 2020). On the one hand, some authors showed that RAP lesions could be characterized by a worse response to intravitreal treatment compared with other forms of nAMD. (Tsai et al., 2017). On the other hand, recent evidence demonstrated that anti-VEGF therapy can provide positive outcomes in RAP eyes, comparable with other types of nAMD (Browning et al., 2019) or even better. (Invernizzi et al., 2019). However, RAP treatment based on intravitreal anti-VEGF therapy alone could prove challenging because of frequent relapses of exudative activity and partial response to this therapy. (Viola et al., 2009). Additionally, in some cases, a more intense intravitreal anti-VEGF treatment could be required. (Rouvas et al., 2012; Gharbiya et al., 2014; Inoue et al., 2014).

On this basis, intravitreal anti-VEGF therapy has been used in combination with photodynamic therapy (PDT) in attempt to achieve a better control of RAP lesions. (Saito et al., 2010; Saito et al., 2012; Saito et al., 2013; Malamos et al., 2018). This combined approach seems to provide promising outcomes in terms of visual gain and macular thickness reduction. (Saito et al., 2010; Saito et al., 2012; Saito et al., 2013; Malamos et al., 2018). However, there is limited evidence as to whether intravitreal anti-VEGF therapy combined with PDT could provide better results compared with intravitreal anti-VEGF therapy alone.

The purpose of the present systematic review with meta-analysis was to collect available evidence on intravitreal anti-VEGF therapy alone or combined with PDT in RAP eyes and to assess whether combining anti-VEGF therapy with PDT could have a synergic effect and lead to better functional and anatomical outcomes.

## 2 Materials and methods

### 2.1 Literature search methods

The study was conducted according to the guidelines of the Preferred Reporting Items for Systematic Reviews and Meta-Analyses (PRISMA) group (PRISMA checklist available in Supplementary Table S1 as Supplementary Material). (Liberati et al., 2009)

We conducted comprehensive searches of PubMed and Embase databases, from January 2009 to 5th May 2022. The electronic search strategy included the terms “retinal angiomatous proliferation,” “RAP,” “type 3 neovascularization,” “choroidal neovascularization,” “anti-vascular endothelial growth factor,” “aflibercept,” “ranibizumab,” “bevacizumab” and “photodynamic therapy,” which were connected by using “and/or” in various combinations. Only articles published in peer-reviewed journals and in English were selected. We also screened reference lists of included studies and review articles focused on similar topics.

### 2.2 Eligibility criteria and outcomes of interest

The following eligibility criteria were considered: 1) to include eyes affected by retinal angiomatous proliferation that were treated with intravitreal anti-VEGF therapy alone (bevacizumab, ranibizumab and aflibercept) and/or in combination with photodynamic therapy; 2) to have a follow-up of at least 12 months; 3) to report visual and/or anatomical outcomes. Case reports and case series with less than 10 cases were excluded. Choroidal neovascular membranes different from RAP were excluded. When clarifications for study eligibility were needed, we contacted study’s authors.

Eyes treated with anti-VEGF therapy alone were included in the anti-VEGF group, while eyes treated with anti-VEGF therapy combined with PDT were included in the combined group.

The primary outcome of interest was the mean change in best corrected visual acuity (BCVA) in the two groups. Mean change in central macular thickness (CMT) on optical coherence tomography (OCT) was considered as secondary outcome. The influence of the number of injections on BCVA and CMT in either group was considered a secondary outcome as well. Central macular thickness referred to the average thickness of the fovea-centered area with 1 mm diameter.

### 2.3 Data collection and risk of bias

Two investigators (MF and IM) evaluated independently the eligibility of identified studies. The same two investigators (MF and IM) analyzed and extracted data from each included study in an independent fashion. A third investigator (VB) was involved in case of disagreement. The following items were collected from each included study: first author, publication year, country, study design, number of eyes, mean age, type of treatment, follow-up. For both the anti-VEGF group and the combined group, the following data were collected: number of eyes, mean age, naïve/non-naïve status, type of anti-VEGF drug and treatment protocol, number of injections, BCVA change, CMT change, follow-up. Information on type of PDT protocol, namely, standard verteporfin PDT, (Bressler, 2001), half-dose and half-fluence PDT, (Reibaldi et al., 2010), was collected for the combined group.

Risk of bias of randomized trials was evaluated by the means of the Cochrane collaboration tool. (Higgins, 2022). Risk of bias assessment for non-randomized studies was based on the Methodological Item for Non- Randomized Studies (MINORS) scale, (Slim et al., 2003), being a ≥9 score at low-to-moderate risk. (Fallico et al., 2020a).

### 2.4 Statistical analysis

For BCVA and CMT change, pooled effect size was investigated through meta-analysis and mean difference (MD) between pre- and post-treatment values was reported along with 95% Confidence Interval (95% CI). The I^2^ index and the Q-statistics were used to measure and test heterogeneity across studies. When a significant heterogeneity was found (I^2^ > 50% and Q-statistics *p* < 0.1), a random effect model was fitted applying the DerSimonian-Laird method. Subgroup analyses were conducted to compare BCVA and CMT outcomes between the anti-VEGF group and the combined groups. Meta-regressions were performed to assess the influence of anti-VEGF number of injections on BCVA and CMT outcomes. Results of the meta-regressions were reported as β coefficient and its standard error (SE). Publication bias was tested using the Egger’s test and by visual inspection of funnel plots’ symmetry. Analyses were conducted on STATA (version 17) and were two-tailed, with a level of statistical significance *α* < 0.05.

## 3 Results

**FIGURE 1:**
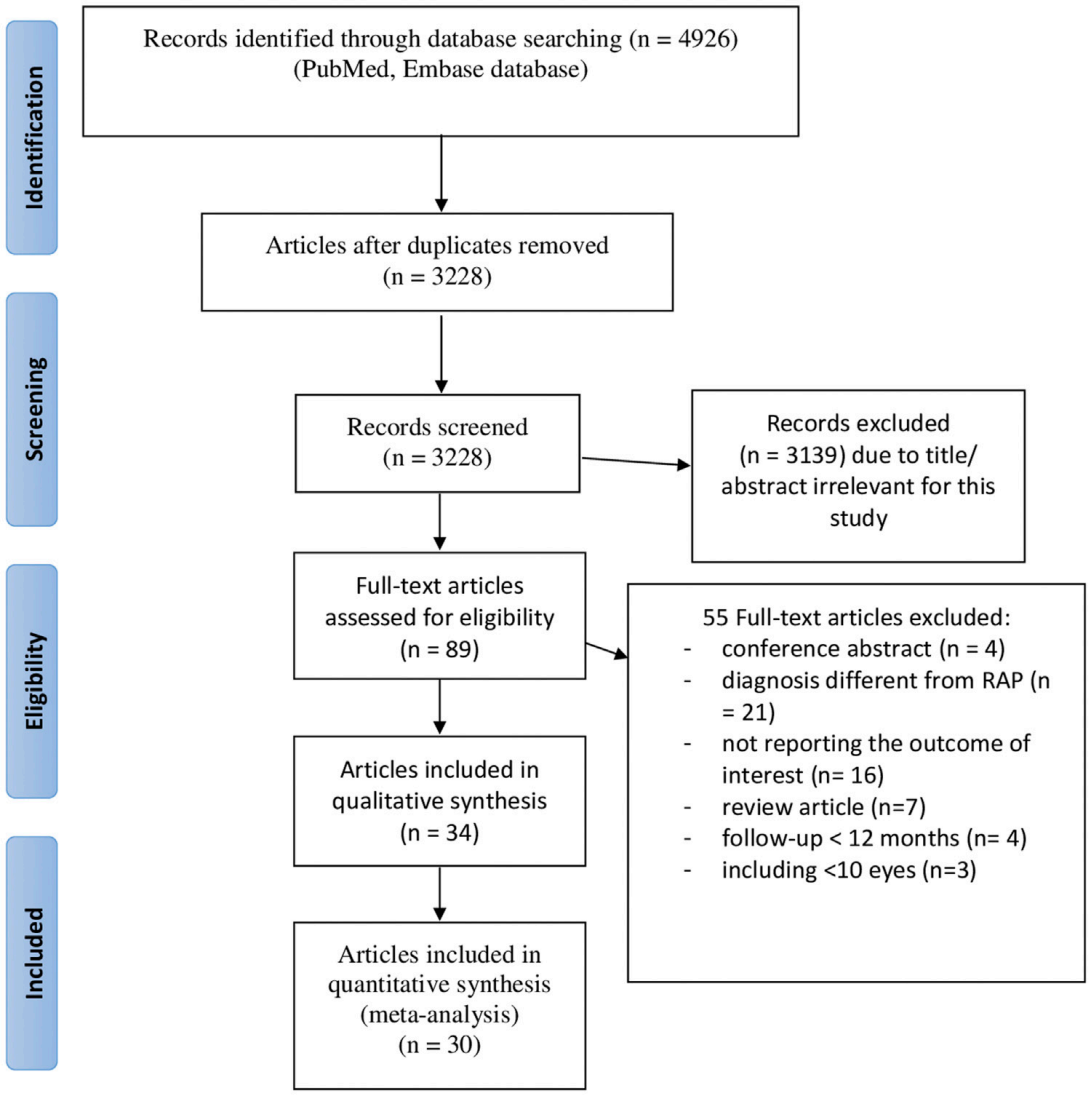
Bow diagram of study selection process.

The flow diagram of the study selection is illustrated in Figure 1. Systematic search identified a total of 4,926 articles, of which 1,698 were duplicates. Titles and abstracts of the remaining 3,228 articles were reviewed for eligibility. A total of 89 articles received a full-text evaluation, of which 55 were excluded because they did not meet inclusion criteria. Thirty-four studies were included.

### 3.1 Characteristics of included studies

Of the 34 included studies, 24 reported on anti-VEGF therapy alone (Engelbert et al., 2009; Montero et al., 2009; Atmani et al., 2010; Hemeida et al., 2010; Reche-Frutos et al., 2011; Parodi et al., 2013; Gharbiya et al., 2014; Inoue et al., 2014; Shin and Yu, 2014; Park and Roh, 2015; Cho et al., 2016; Matsumoto et al., 2016; Kim et al., 2017a; Kim et al., 2017b; Hata et al., 2017; Kim et al., 2018; Browning et al., 2019; Invernizzi et al., 2019; Kim et al., 2019; Maruyama-Inoue et al., 2019; Arias et al., 2020; Ernest et al., 2020; Kim, 2020; Kim et al., 2020), 8 reported on anti-VEGF therapy combined with PDT (Saito et al., 2010; Lee et al., 2011; Nakano et al., 2012; Saito et al., 2012; Saito et al., 2013; Seidel et al., 2013; Saito et al., 2016; Malamos et al., 2018) and 2 studies compared anti-VEGF therapy alone *versus* combined with PDT. (Rouvas et al., 2012; Arias et al., 2016).

#### 3.1.1 Anti-VEGF group

Cohorts from 26 studies were included in the anti-VEGF group, with a total of 1,221 eyes. Characteristics of included studies are shown in Table 1. Publication year ranged from 2009 to 2020. Of 26 studies, 15 were retrospective, (Engelbert et al., 2009; Montero et al., 2009; Hemeida et al., 2010; Inoue et al., 2014; Park and Roh, 2015; Cho et al., 2016; Matsumoto et al., 2016; Kim et al., 2017a; Kim et al., 2017b; Hata et al., 2017; Kim et al., 2018; Kim et al., 2019; Maruyama-Inoue et al., 2019; Kim, 2020; Kim et al., 2020), 8 were prospective, (Atmani et al., 2010; Reche-Frutos et al., 2011; Gharbiya et al., 2014; Shin and Yu, 2014; Browning et al., 2019; Invernizzi et al., 2019; Arias et al., 2020; Ernest et al., 2020), and 3 were randomized trials. (Rouvas et al., 2012; Parodi et al., 2013; Arias et al., 2016). Two randomized trials compared anti-VEGF therapy alone *versus* PDT combined with anti-VEGF therapy, (Rouvas et al., 2012; Arias et al., 2016), while Parodi et al. (Parodi et al., 2013) compared ranibizumab *versus* bevacizumab. In all studies RAP diagnosis was based on fluorescein and indocyanine green angiography. Nine studies reported on ranibizumab only, (Atmani et al., 2010; Reche-Frutos et al., 2011; Rouvas et al., 2012; Inoue et al., 2014; Shin and Yu, 2014; Park and Roh, 2015; Arias et al., 2016; Kim et al., 2017a; Kim et al., 2017b), 4 studies on aflibercept only (Matsumoto et al., 2016; Browning et al., 2019; Arias et al., 2020; Ernest et al., 2020) and one study on bevacizumab only; (Montero et al., 2009); 5 studies reported on both ranibizumab and bevacizumab, (Engelbert et al., 2009; Hemeida et al., 2010; Parodi et al., 2013; Gharbiya et al., 2014; Cho et al., 2016), 3 studies on both ranibizumab and aflibercept (Hata et al., 2017; Kim et al., 2018; Maruyama-Inoue et al., 2019) and 4 studies on all three anti-VEGF agents. (Invernizzi et al., 2019; Kim et al., 2019; Kim, 2020; Kim et al., 2020). Twenty-two studies included only naïve eyes (Engelbert et al., 2009; Montero et al., 2009; Atmani et al., 2010; Rouvas et al., 2012; Parodi et al., 2013; Gharbiya et al., 2014; Inoue et al., 2014; Shin and Yu, 2014; Park and Roh, 2015; Cho et al., 2016; Matsumoto et al., 2016; Kim et al., 2017a; Kim et al., 2017b; Hata et al., 2017; Kim et al., 2018; Browning et al., 2019; Invernizzi et al., 2019; Maruyama-Inoue et al., 2019; Arias et al., 2020; Ernest et al., 2020; Kim, 2020; Kim et al., 2020), 2 studies included non-naïve eyes, (Reche-Frutos et al., 2011; Arias et al., 2016), and two studies did not provide information about previous treatment. (Hemeida et al., 2010; Kim et al., 2019). In all studies but one (Engelbert et al., 2009; Montero et al., 2009; Atmani et al., 2010; Reche-Frutos et al., 2011; Parodi et al., 2013; Gharbiya et al., 2014; Inoue et al., 2014; Shin and Yu, 2014; Park and Roh, 2015; Cho et al., 2016; Matsumoto et al., 2016; Kim et al., 2017a; Kim et al., 2017b; Hata et al., 2017; Kim et al., 2018; Browning et al., 2019; Invernizzi et al., 2019; Kim et al., 2019; Maruyama-Inoue et al., 2019; Arias et al., 2020; Ernest et al., 2020; Kim, 2020; Kim et al., 2020), a loading phase of 3 monthly injections was administered at baseline, followed by the selected regimen. Hemeida et al. (Hemeida et al., 2010) gave only one injection at baseline, which was followed by a pro re nata (PRN) protocol. A PRN was adopted in 20 trials (Montero et al., 2009; Atmani et al., 2010; Hemeida et al., 2010; Reche-Frutos et al., 2011; Rouvas et al., 2012; Parodi et al., 2013; Gharbiya et al., 2014; Inoue et al., 2014; Shin and Yu, 2014; Park and Roh, 2015; Arias et al., 2016; Cho et al., 2016; Kim et al., 2017a; Kim et al., 2017b; Kim et al., 2018; Kim et al., 2019; Maruyama-Inoue et al., 2019; Kim, 2020; Kim et al., 2020), while 3 studies used a treat and extend regimen (Engelbert et al., 2009; Matsumoto et al., 2016; Arias et al., 2020) and 2 a fixed regimen with bimonthly injections. (Browning et al., 2019; Ernest et al., 2020). Hata et al. (Hata et al., 2017) used two different treatment protocols according to the anti-VEGF agent: a PRN regimen was used in the ranibizumab arm, while a fixed bimonthly regimen was used in aflibercept arm. In 19 out of 20 studies which followed a PRN regimen, retreatment was performed with a single intravitreal injection, while in one study (Rouvas et al., 2012) retreatment consisted of 3 more monthly intravitreal injections.

**TABLE 1:** Characteristics of included studies in the anti-VEGF monotherapy group.

Author and year	Design	Anti-VEGF drug	Eyes (n)	Gender (M/W)	Age mean (±SD, years/range)	Follow-up (months)	Anti VEGF regimen	RAP stage (n)
Montero et al. (2009)	Retrospective	Bevacizumab	26 naive	9 /15	78 ± 8	12	3 IV monthly + PRN (followed monthly)	-14 stage 2
								-12 stage 3
Engelbert et al. (2009)	Retrospective	Bevacizumab/Ranibizumab	11 naive	3/8	85 (range, 71–92)	12	3 IV monthly + Treat and extend*	-
Atmani et al. (2010)	Prospective	Ranibizumab	29 naive	7/19	78.2 ± 6.7 (range 66-90)	12	3 IV monthly + PRN (follow up interval not specified)**	stage 2/3
Parodi et al. (2013)	Prospective randomized	Bevacizumab/ Ranibizumab	50 naive	21/29	73 ± 7.5	12	3 IV monthly + PRN (followed monthly)	Bevacizumab
								-14 stage1
								-12 stage 2B Ranibizumab
								-14 stage 1
								-10 stage 2B
Reche-Frutos et al. (2011)	Prospective	Ranibizumab	53 (31 naive)	16/37	81.91 ± .3	53	3 IV monthly + PRN (followed monthly)	-21 stage 2A
								-18 stage 2B
								-14 Stage 3
Rouvas et al. (2012)	Randomized controlled trial	Ranibizumab	13 naive	5/8	76.87	36	3 IV monthly + PRN	-10 stage 2
							-Retreatment: 3 IV monthly	-3 stage3
Shin and Yu (2014)	Prospective	Ranibizumab	31 naive	6/25	70.4 ± 6.5	24	3 IV monthly + PRN (followed monthly)	-5 stage 1
								-12 stage 2
								-14 stage 3
Gharbiya et al. (2014)	Prospective	Bevacizumab/Ranibizumab	21 naive	5/14	74.5 ± 9.6	36	3 IV monthly + PRN (followed monthly)	-

Author and year	Design	Anti-VEGF drug	Eyes (n)	Gender (M/W)	Age mean (±SD, years/range)	Follow-up (months)	Anti VEGF regimen	RAP stage (n)
Inoue et al. (2014)	Retrospective	Ranibizumab	17 naive	4/10	80.5 ± 4.7	36	3 IV monthly + PRN (followed monthly)	-1 stage 1
								10stage 2
								-6 stage 3
Park and Roh (2015)	Retrospective	Ranibizumab	41 naive	16/25	67.09 ± 11.76	12	3 IV monthly + PRN (followed monthly)	-8 stage 1
								-17 stage 2
								-16 stage 3
Cho et al. (2016)	Retrospective	Bevacizumab/Ranibizumab	38 naive	20/18	74.3 ± 7.5	36	3 monthly IV + PRN (followed monthly)	-4 stage 1
								-24 stage 2
								-10 stage 3
Arias et al. (2016)	Randomized controlled trial	Ranibizumab/Ranibizumab + PDT	10 no naive	3/7	79.5 ± 8.0	12	3 IV monthly + PRN (followed monthly)	-2 stage 1
								-6 stage 2
								-2 stage 3
Matsumoto et al. (2016)	Retrospective	Aflibercept	17 naive	6/11	76.9	12	3 monthly IV + Treat and extend°	-8 stage 1
								-1 stage 2 without PED
								-7 stage 2 with PED
								-1 stage 3
Hemeida et al. (2010)	Retrospective	Bevacizumab/Ranibizumab	20	6/9	85.8 ± 4.54	24	1 IV baseline + PRN (followed monthly)	-
Kim et al. (2017a)	Retrospective	Ranibizumab	42 naive	13/29	75.5 ± 5.8	12	3 IV monthly + PRN ***	-
Kim et al. (2017b)	Retrospective	Ranibizumab	38 naive	4/15	75.8 ± 7.7	12	3 IV monthly + PRN ***	-17 stage 1
								-21 stage 2
Hata et al. (2017)	Retrospective	Ranibizumab/Aflibercept	41 naive	16/30	82.2 ± 6.6	27.6 ± 15.5	Ranibizumab 3 IV monthly + PRN (followed monthly)	-.4 stage 1
								-15 stage 2
							Aflibercept 3 IV monthly + fixed (bimonthly IV)	-27 stage 3

Author and year	Design	Anti-VEGF drug	Eyes (n)	Gender (M/W)	Age mean (±SD, years/range)	Follow-up (months)	Anti VEGF regimen	RAP stage (n)
Kim et al. (2018)	Retrospective	Ranibizumab /Aflibercept	42 naive	6/34	76.3 ± 6.4	12	Ranibizumab/ Aflibercept 3 monthly IV + PRN ***	-14 stage 2
								-26 stage 3
Invernizzi et al. (2019)	Prospective	Bevacizumab/Ranibizumab /Aflibercept	157 naive	46/111	83.1 ± 6.4, (65–96)	12	§	-
Ernest et al. (2020)	Prospective	Aflibercept	14 naive	9/5	71 ± 9	12	3 monthly IV +fixed (bimonthly IV)	-7 Stage 2
								-7 Stage 3
Maruyama-Inoue et al. (2019)	Retrospective	Ranibizumab/Aflibercept	85 naïve	21/40	84.0 ± 6.7	36	68 eyes	-8 stage 1
							3 monthly IV + PRN (followed monthly or bimonthly)	-53 stage 2
							17 eyes	-24 stage 3
							Fixed monthly o bimonthly	
Browning et al. (2019)	Prospective	Aflibercept	46 naïve	12/34	81.5	24	3 IV monthly + fixed (bimonthly IV)	-3 stage 1
								-9 stage 2
								-34 stage 3
Kim et al. (2019)	Retrospective	Ranibizumab/Aflibercept (retreatment with Ranibizumab/Aflibercept or Bevacizumab)	137	38/99	74.9 ± 5.9	42.4±18.9	3 IV monthly + PRN ***	-32 stage 2
								-105 stage 3
Arias et al. (2020)	Prospective,multicenter trial	Aflibercept	32 naive	10/22	78.2 ± 7.7	12	3 IV monthly + Treat and Extend°°	-

Author and year	Design	Anti-VEGF drug	Eyes (n)	Gender (M/W)	Age mean (±SD, years/range)	Follow-up (months)	Anti VEGF regimen	RAP stage (n)
Kim et al. (2020)	Retrospective	Ranibizumab/Aflibercept Retreatment with Ranibizumab, Aflibercept or Bevacizumab	195 naive	42/153	75.7 ± 6.0	47.5 ± 20.7	3 IV monthly + PRN (followed monthly) or switch treat and extend	-43 stage 2
								-152 stage 3
Kim (2020)	Retrospective	Ranibizumab/Aflibercept Retreatment with Ranibizumab, Aflibercept or Bevacizumab	17 naive	2/15	75.4 ± 6.2	39.7 ± 10.9	3 IV monthly + PRN (followed monthly)	-2 stage 2
								-15 stage 3

N: number; M/W: men/women; SD: standard deviation; IV: intravitreal; RAP: retinal angiomatous proliferation; PRN: *pro re nata*

*Treat and Extend: at least 3 monthly injections followed by continued treatment at intervals increasing by 2 weeks per visit once visual acuity was stable.

**Further treatments were given if any of the following changes applied: best corrected visual acuity (BCVA) loss of at least five letters associated with fluid within the macula as evaluated by OCT, central macular thickness (CMT) increase of at least 100 mm, and/or persistence of fluid within the macula as evaluated by OCT, new onset macular haemorrhages, persistence of leakage from the lesions on fluorescein angiography.

°In the maintenance phase, the interval of injections is extended by 2 weeks if there is no exudative change, The scheduled treatment interval is extended to a maximum of 12 weeks in the current study.

***After a loading phase, patients were scheduled to attend the hospital every 1 month to 2 months. In some of the cases without long-term recurrence, the follow-up period was extended up to 3 months at the discretion of the treating physician.

§Treatment decisions, such as the choice of drug and frequency and timing of treatment, were entirely at the discretion of the practitioner in consultation with the patient, thereby reflecting real-world practice. Only eyes that had received at least three injections in the first year of treatment were included in the study.

°°Retreatment (initially scheduled at Weeks 12–14) was extended by 2 weeks per visit (in relation to the period since the last visit) to a maximum of 12 weeks if no evidence of exudative disease activity was observed. If there were signs of exudative disease, the patient was retreated and the next visit was 4 weeks later.

Follow-up period ranged from 12 months to 48 months. Twenty-two studies (Engelbert et al., 2009; Montero et al., 2009; Atmani et al., 2010; Hemeida et al., 2010; Reche-Frutos et al., 2011; Parodi et al., 2013; Inoue et al., 2014; Shin and Yu, 2014; Park and Roh, 2015; Arias et al., 2016; Cho et al., 2016; Matsumoto et al., 2016; Kim et al., 2017a; Kim et al., 2017b; Hata et al., 2017; Kim et al., 2018; Browning et al., 2019; Invernizzi et al., 2019; Kim et al., 2019; Arias et al., 2020; Ernest et al., 2020; Kim et al., 2020) provided data on 12-month follow-up, while 4 studies (Rouvas et al., 2012; Gharbiya et al., 2014; Maruyama-Inoue et al., 2019; Kim, 2020) did not report 12-month outcomes, providing only outcomes at 24 months or longer.

#### 3.1.2 Anti-VEGF combined with PDT

Cohorts from 10 studies were included in the combined group, with a total of 159 eyes. Characteristics of included studies are shown in Table 2. Publication year ranged from 2010 to 2018. Of these 10 studies, 5 were retrospective (Saito et al., 2010; Nakano et al., 2012; Saito et al., 2012; Saito et al., 2013; Saito et al., 2016), 3 were prospective (Lee et al., 2011; Seidel et al., 2013; Malamos et al., 2018) and two were randomized trials. (Rouvas et al., 2012; Arias et al., 2016). The two randomized trials compared anti-VEGF therapy alone *versus* PDT combined with anti-VEGF therapy. In all studies, RAP diagnosis was based on fluorescein and indocyanine green angiography. PDT was combined with intravitreal ranibizumab in 8 studies (Lee et al., 2011; Nakano et al., 2012; Rouvas et al., 2012; Saito et al., 2012; Seidel et al., 2013; Arias et al., 2016; Saito et al., 2016; Malamos et al., 2018), with bevacizumab in one study (Saito et al., 2010) and with either ranibizumab or bevacizumab in another one. (Saito et al., 2013). Seven studies included only naïve eyes (Saito et al., 2010; Lee et al., 2011; Nakano et al., 2012; Rouvas et al., 2012; Saito et al., 2012; Saito et al., 2013; Saito et al., 2016), 3 studies included non-naïve eyes. (Seidel et al., 2013; Arias et al., 2016; Malamos et al., 2018). A loading phase of 3 monthly injections was administered in 5 studies (Lee et al., 2011; Rouvas et al., 2012; Saito et al., 2012; Arias et al., 2016; Saito et al., 2016), while a single intravitreal injection was give as loading phase in the remaining 5 studies. (Saito et al., 2010; Nakano et al., 2012; Saito et al., 2013; Seidel et al., 2013; Malamos et al., 2018). A PRN regimen was used in all studies. (Saito et al., 2010; Lee et al., 2011; Nakano et al., 2012; Rouvas et al., 2012; Saito et al., 2012; Saito et al., 2013; Seidel et al., 2013; Arias et al., 2016; Saito et al., 2016; Malamos et al., 2018). Retreatment was done with either 3 more 4-weekly injections (Lee et al., 2011; Rouvas et al., 2012; Saito et al., 2013) or a single intravitreal injection. (Saito et al., 2010; Nakano et al., 2012; Saito et al., 2012; Seidel et al., 2013; Arias et al., 2016; Saito et al., 2016; Malamos et al., 2018). As regards PDT, a standard-dose and standard-fluence PDT was used in 8 studies, (Saito et al., 2010; Lee et al., 2011; Nakano et al., 2012; Rouvas et al., 2012; Saito et al., 2012; Saito et al., 2013; Seidel et al., 2013; Saito et al., 2016), in one study a half-dose and standard-fluence PDT was performed, (Malamos et al., 2018), Arias et al. did not report information on PDT parameters. (Arias et al., 2016). In 8 studies PDT was performed both at baseline and at each retreatment (Saito et al., 2010; Lee et al., 2011; Nakano et al., 2012; Rouvas et al., 2012; Saito et al., 2012; Saito et al., 2013; Arias et al., 2016; Saito et al., 2016), while in 2 studies PDT was performed only at baseline. (Seidel et al., 2013; Malamos et al., 2018). Follow-up period ranged from 12 months to 38 months. All studies but one provided data on 12-month outcomes. (Saito et al., 2010; Lee et al., 2011; Nakano et al., 2012; Saito et al., 2012; Saito et al., 2013; Seidel et al., 2013; Arias et al., 2016; Saito et al., 2016; Malamos et al., 2018). Only Rouvas et al. (Rouvas et al., 2012) did not report on 12-month follow-up, providing outcomes at 36 months only.

**TABLE 2:** Characteristics of included studies in the combined group.

Author and year	Design	Anti-VEGF drug	Eyes (n)	Gender (M/W)	Age mean (±SD, years/range)	Follow-up (months)	Anti VEGF regimen	Stage	PDT dose/fluence	PDT regimen
Saito et al. (2010)	Retrospective	Bevacizumab	13 naive	8/3	78.3 (range 63–89)	12	baseline +PRN	-5 stage 2 without PED	standard dose and standard fluence	baseline + PRN
								-6 stage 2 with PED		
								-2 stage 3		
Lee et al. (2011)	Prospective	Ranibizumab	10 naive	2/7	76 (range 65–87)	12	3 IV monthly + PRN		standard dose and standard fluence	baseline + PRN
							-Retreatment: 3 more monthly IV			
Saito et al. (2012)	Retrospective	Ranibizumab	20 naive	8/8	84.8 ± 4.8	12	3 IV monthly + PRN	-11 stage 2 without PED	standard dose and standard fluence	baseline + PRN
								-7 stage 2 with PED		
							-Retreatment: 1 IV	-2 stage 3		
Rouvas et al. (2012)	Randomized controlled trial	Ranibizumab	13	4/9	77.12	36	3 IV monthly + PRN	13 stage 2	standard dose and standard fluence	baseline + PRN
							-Retreatment: 3 IV monthly			
Nakano et al. (2012)	Retrospective	Ranibizumab	11 naive	4/7	80.3 ± 7.2	12	1 IV at baseline +PRN	-3 stage 1	standard dose and standard fluence	baseline + PRN
							-Retreatment: 1 IV	-5 stage 2		
								-3 stage 3		

Author and year	Design	Anti-VEGF drug	Eyes (n)	Gender (m/w)	Age mean (±SD, years/range)	Follow-up (months)	Anti VEGF regimen	Stage	PDT dose/fluence	PDT regimen
Saito et al. (2013)	Retrospective	Bevacizumab/Ranibizumab.	13 naive	7/5	77.0 ± 9.5	24	1 IV Bevacizumab at baseline + PRN	-7 stage 1 without PED	Standard dose and standard fluence	Baseline + PRN
							-Retreatment:	-5 stage 2 with PED		
							Before February 2009: 1 IV Bevacizumab	-1 stage 3		
							After March 2009: 3 monthly Ranibizumab			
Seidel et al. (2013)	Prospective	Ranibizumab	15 naive	4/10	79.7 ± 4.7	12	1 IV at baseline + PRN	-7 stage 1	Standard dose and standard fluence	Baseline only
							-Retreatment:	-4 stage 2a		
							1 IV	-4 stage 2b		
Saito et al. (2016)	Retrospective	Ranibizumab	37 naive	12/19	82.0 ± 6.3	24	3 monthly IV + PRN	-2 stage 1	Standard dose and standard fluence	Baseline + PRN 1
							-Retreatment: 1 IV	-20 stage 2 without PED;		
								-13 stage 2 with PED - 2 stage 3		
Arias et al. (2016)	Randomized controlled trial	Ranibizumab	10	6/4	79.2 ± 3.7	12	3 monthly IV +PRN	-3 stage 1	-	Baseline + PRN (followed monthly) *
			7 naive				-Retreatment: 1 IV (followed monthly)*	-5 stage 2		
								-2 stage 3		
Malamos et al. (2018)	Prospective	Ranibizumab	17	-	80.7	24.7	1 IV at baseline + PRN	-3 stage 1	Half dose standard fluence	Baseline only
			13 naive				-Retreatment: 1 IV	-4 stage 2		
								-8 stage 3		

N, number; M/W: men/women; SD, standard deviation; IV, intravitreal; RAP, retinal angiomatous proliferation; PRN, *pro re nata*; PDT, photodynamic therapy; PED, pigmented epithelium detachment.

*All retreatments in group B consisted of combined therapy of a single intravitreal injection of ranibizumab and PDT with verteporfin. In addition, in group B, ranibizumab 0.5 mg could be administered in monotherapy as rescue therapy, if necessary.

### 3.2 Quality assessment

Risk of bias assessment for randomized studies is illustrated in Supplementary Figures S1, S2. Random sequence generation was deemed as low risk in one trial (Parodi et al., 2013) and unclear risk in 2 trials. (Rouvas et al., 2012; Arias et al., 2016). Risk of allocation concealment bias was unclear for all randomized trials. (Rouvas et al., 2012; Parodi et al., 2013; Arias et al., 2016). Risk of both performance bias and detection bias was judged high in one trial (Rouvas et al., 2012) and unclear for the two other. (Parodi et al., 2013; Arias et al., 2016). Attrition bias was considered low in all randomized trials. (Rouvas et al., 2012; Parodi et al., 2013; Arias et al., 2016). Reporting bias was judged as low risk in 2 trials, (Parodi et al., 2013; Arias et al., 2016), while Rouvas’s study (Rouvas et al., 2012) was considered as high risk because the primary outcome of the trial was not pre-specified and the outcomes of interest of the present systematic review were not reported. Risk for other bias was unclear in 2 trials, (Rouvas et al., 2012; Parodi et al., 2013), while Arias’s study (Arias et al., 2016) was judged as high risk because it failed to achieve the sample size that was initially planned. The MINORS scale assessment for non-randomized studies is shown in Supplementary Table S2, with all studies achieving a ≥10 score.

The inspection of funnel plots did not allow to completely exclude the presence of publication bias, especially for the CMT outcome (Supplementary Figures S3, S4).

### 3.3 Best corrected visual acuity change

**FIGURE 2:**
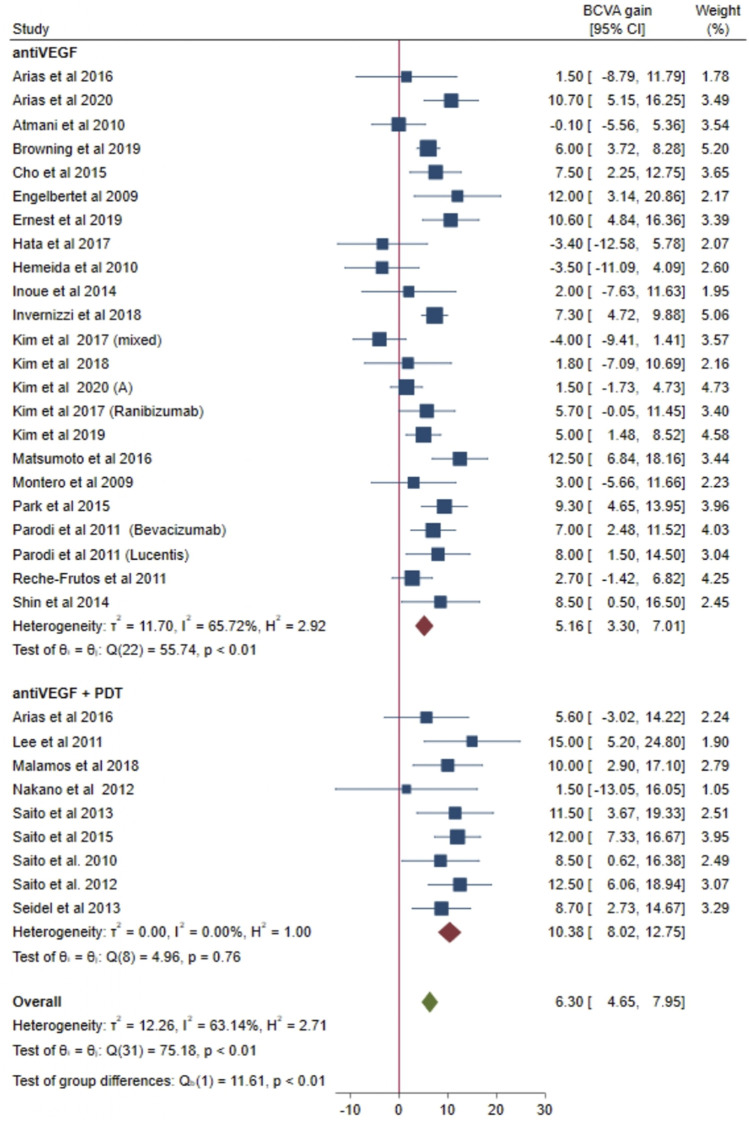
Comparison of best corrected visual acuity (BCVA) gain after 1 year of treatment with anti-VEGF alone or in combination with photodynamic therapy (PDT).

Data from 22 and 9 studies were pooled together to explore mean visual change at 12 months in the anti-VEGF group and in the combined group, respectively (Figure 2). Overall, considering both groups together, a mean gain of 6.30 letters was evident at 12 months (95% CI = 4.65–7.95). A high heterogeneity was found when considering both groups together (I^2^ = 63.14%; *p* < 0.01). In the anti-VEGF group, a mean gain of 5.16 letters was shown at 12 months (95% CI = 3.30–7.01). A significant heterogeneity was found across studies included in the anti-VEGF group (I^2^ = 65.72%; *p* < 0.01). In the combined group, a mean gain of 10.38 letters was found at 12 months (95% CI = 8.02–12.75). Heterogeneity was low in the combined group (I^2^ = 0%; *p* = 0.76). Of note, visual gain in the combined group was higher compared with the anti-VEGF group (10.38 *versus* 5.16, respectively) and 95% CI of the combined group does not overlap with those of the anti-VEGF group. Accordingly, the test of group differences revealed that 12-month visual gain in the combined group was significantly greater compared with the anti-VEGF group (*p* < 0.01).

### 3.4 Central macular thickness change

**FIGURE 3:**
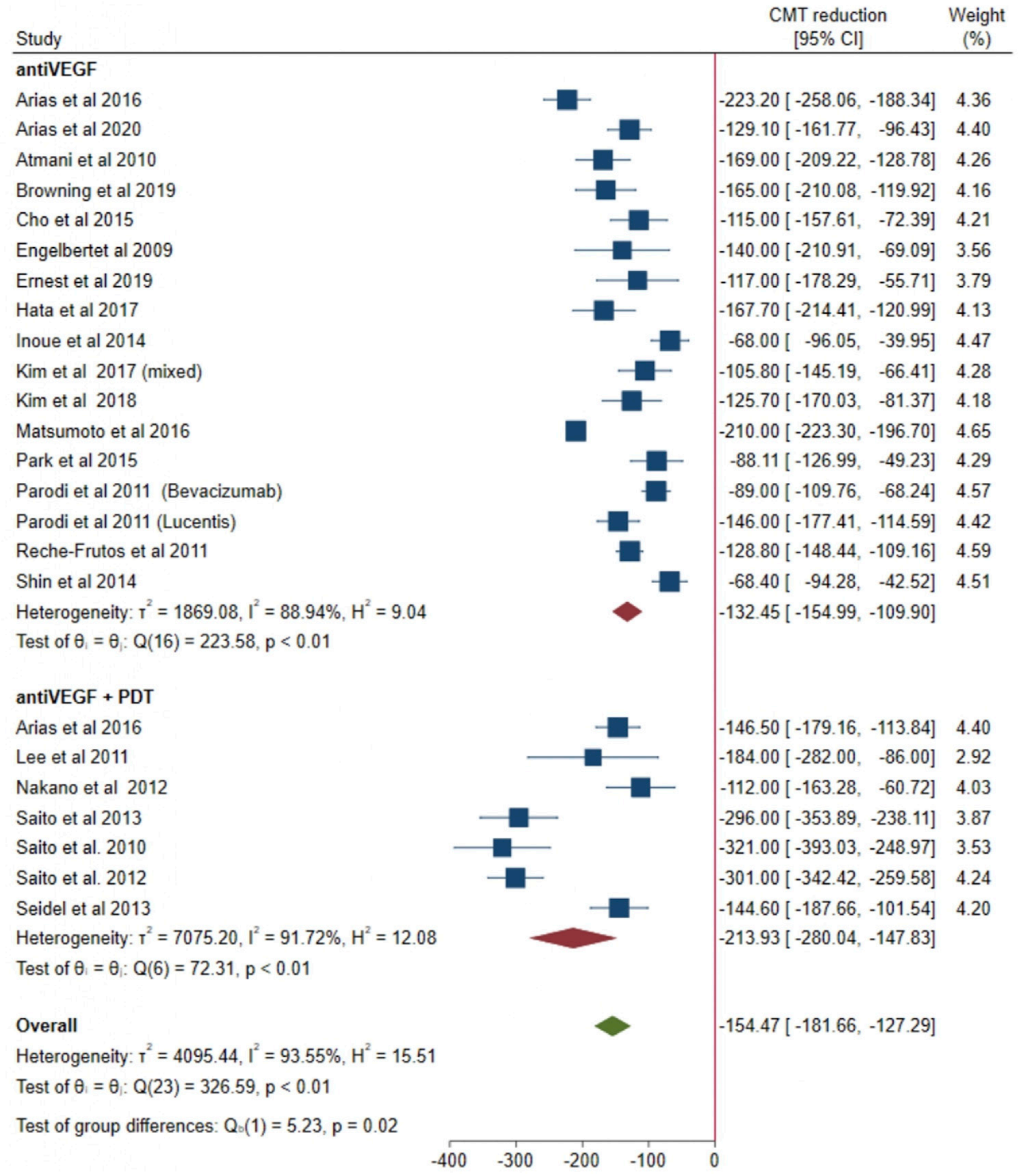
Comparison of central macular thickness (CMT) reduction after 1 year of treatment with anti-VEGF alone or in combination with photodynamic therapy (PDT).

Data from 16 and 7 studies were pooled together to explore mean CMT change at 12 months in the anti-VEGF group and in the combined group, respectively (Figure 3). Overall, considering both groups together, a mean CMT reduction of 154.47 µm was shown at 12 months (95% CI = from −181.66 to −127.29). A high heterogeneity was found when considering both groups together (I^2^ = 93.55%; *p* < 0.01). In the anti-VEGF group, a mean CMT reduction of 132.45 µm was found at 12 months (95% CI = from −154.99 to −109.90). A significant heterogeneity was shown across studies included in the anti-VEGF group (I^2^ = 88.94%; *p* < 0.01). In the combined group, a mean CMT reduction of 213.93 µm was evident at 12 months (95% CI = from −280.04 to −147.83). Heterogeneity was high in the combined group as well (I^2^ = 91.72%; *p* < 0.01). Of note, at 12 months a greater CMT reduction was shown in the combined group compared with the anti-VEGF group (−213.93 µm *versus* −132.45 µm, respectively) and 95% CI of the combined group does not overlap with those of the anti-VEGF group. Accordingly, the test of group differences demonstrated a significantly greater CMT decrease in the combined group compared with the anti-VEGF group (*p* = 0.02).

### 3.5 Influence of injection number

The average number of injections over a 12-month follow-up was higher in the anti-VEGF group compared with the combined group: a mean of 4.9 injections (95% CI = 4.2–5.6) were administered in the anti-VEGF group while a mean of 2.8 injections (95% CI = 1.3–4.4) were administered in the combined group (*p* = 0.02).

Meta-regression analyses showed no influence of injection number on visual and CMT outcomes in either group and overall considering both groups together (Table 3).

**TABLE 3:** Meta-regressions showing the effect of the number of injections on BCVA and CMT outcomes.

Outcome and treatment group	Effect of the number of injections, β (standard error)	*p*-value
BCVA		
Overall	0.16 (0.49)	0.741
anti-VEGF	0.74 (0.61)	0.216
anti-VEGF combined with PDT	0.02 (1.25)	0.984
CMT		
Overall	10.32 (8.07)	0.201
anti-VEGF	−11.34 (8.15)	0.164
anti-VEGF combined with PDT	18.16 (30.04)	0.546

Footnote: BCVA, best corrected visual acuity; CMT, central macular thickness; PDT, photodynamic therapy.

## 4 Discussion

This meta-analysis investigated functional and anatomical outcomes of intravitreal anti-VEGF therapy alone or combined with PDT in eyes with RAP, comparing these two different therapeutic options. In summary, our findings showed that anti-VEGF therapy combined with PDT provided a better visual gain and a greater CMT reduction compared with anti-VEGF therapy alone over a 12-month follow-up.

Treatment of RAP lesions could represent a challenge for medical retina physicians because this type of neovascular membranes may show a poor or incomplete response to traditional intravitreal anti-VEGF drugs. (Viola et al., 2009; Tsai et al., 2017). This behavior could be related to the anatomical and pathogenetic characteristics of RAP. (Ghazi and Conway, 2005; Haj Najeeb et al., 2021).

Many published studies have shown that intravitreal anti-VEGF therapy is effective in improving visual outcomes and in reducing vascular leakage and retinal oedema. (Costagliola et al., 2007; Viola et al., 2009; Tsai et al., 2017). Even if recent studies showed a better response to anti-VEGF drugs of RAP lesions when compared to other forms of neovascularization, (Browning et al., 2019; Invernizzi et al., 2019), RAP lesions, in some cases, could require an intense and prolonged treatment due to frequent recurrence of membrane activity. (Rouvas et al., 2012; Saito et al., 2012; Gharbiya et al., 2014; Inoue et al., 2014). Additionally, this type of neovascular membrane has been shown to remain active in most patients on a long-term follow-up. (Costagliola et al., 2007; Gupta et al., 2010; Tsai et al., 2017). Different anti-VEGF agents have been used for RAP treatment with different therapeutic regimens, such us fixed, as needed (pro re nata) and treat-and-extend. (Tsai et al., 2017; Fallico et al., 2020b).

In this scenario, a combined therapy with intravitreal anti-VEGF agents and verteporfin PDT could offer advantages over anti-VEGF monotherapy, slowing or completely blocking the neovascularization process. (Tsai et al., 2017).

The mechanism of action of PDT is based on the activation of verteporfin by a light source with subsequent release of free radicals in the treatment site, specifically in the choriocapillaris. (Bressler, 2001). This process leads to endothelial cell damage and choriocapillaris hypoperfusion. The treatment is highly selective and photoreceptors are spared. (Boscia et al., 2006). After the advent of anti-VEGF therapy, the role of PDT has been significantly downsized. Variations of standard PDT protocol have been introduced in order to reduce the risk of persistent choriocapillaris hypoperfusion and RPE changes. (Reibaldi et al., 2010). Currently, the most commonly adopted protocols are either half-dose PDT or half-fluence PDT, which are mainly used for the treatment of chronic central serous chorioretinopathy (CSC). (Reibaldi et al., 2010). Photodynamic therapy has also been combined with intravitreal therapy for the treatment of choroidal neovascular membranes, including RAP lesions and polypoidal choroidal vasculopathy. (Lim et al., 2020).

Type 3 neovascular membranes are considered as “high-flow” lesions. The rationale of a combined therapy (PDT plus intravitreal anti-VEGFs) lies in a synergic mechanism of action of these two therapeutic approaches. In fact, PDT could induce complete occlusion of the retinal–retinal anastomosis, while intravitreal anti-VEGF therapy could counteract the release of VEGF caused by the PDT-related hypoxia in the choriocapillaris. (Saito et al., 2010; Seidel et al., 2013).

Saito et al. demonstrated a complete occlusion of the retinal–retinal anastomosis in 89.5% of RAP cases treated with this combined therapy. (Saito et al., 2012). However, the evidence of supporting combined approach in RAP treatment is mostly based on small-sized retrospective studies. Furthermore, only two randomized trials compared intravitreal anti-VEGF therapy alone *versus* intravitreal anti-VEGF therapy combined with PDT in RAP eyes. (Rouvas et al., 2012; Arias et al., 2016). Both of these trials were small-sized with less than 15 eyes for each treatment arm. (Rouvas et al., 2012; Arias et al., 2016). Additionally, results of these randomized trials are controversial. On the one hand, Rouvas et al. did not demonstrate any improvement in visual and anatomical outcomes following combined therapy after a 3-year follow-up. (Rouvas et al., 2012). On the other hand, Arias et al. reported a better visual gain in eyes receiving combined therapy, but failed to show any statistical significance. (Arias et al., 2016).

To the best of our knowledge, no previous systematic review has compared the visual and anatomical outcome of combined therapy of anti-VEGF plus PDT *versus* intravitreal anti-VEGF therapy alone in RAP eyes. Tsai et al. (Tsai et al., 2017) performed a review focused on diagnostic and treatment options for RAP. Besides, the authors conducted a meta-analysis of 9 included studies exploring mean change in visual acuity and central foveal thickness, but no comparison between different treatment approaches was made. (Tsai et al., 2017).

Our analyses revealed a better visual improvement in eyes treated with anti-VEGF therapy combined with PDT. Mean visual gain in the combined group was was more than two-fold higher compared with the mean gain of the anti-VEGF monotherapy group. Looking at 95% confidence intervals, the minimal improvement in the combined group (7.66 letters) was yet superior to the maximum improvement in the anti-VEGF alone group (6.56 letters), confirming that the combined approach yielded better visual results.

With regard to macular thickness, the results of the two randomized trials showed a comparable final CMT between eyes treated with anti-VEGF monotherapy and eyes treated with anti-VEGF therapy combined with PDT. (Rouvas et al., 2012; Arias et al., 2016). Conversely, our analyses on 12-month CMT outcome revealed a greater reduction of macular thickness in the combined group compared to the anti-VEGF monotherapy group.

Our findings showed that combined therapy with PDT and intravitreal anti-VEGFs could yield better outcomes with a lower number of injections, thanks to their different and synergic mechanism of action.

This may also have a positive impact on the financial burden, on the anxiety of patients and reduce the risk of injection-related complications and side effects. (Reibaldi et al., 2020; Reibaldi et al., 2022).

In the present meta-analysis, combined therapy has also shown a good safety profile despite PDT being used in all studies except for one with standard dose and fluence.

In light of a widespread use of PDT at lower dose and fluence, further studies are needed to investigate efficacy and safety of a combined approach using modified PDT protocols (half dose or half fluence) for RAP lesions.

In the present meta-analysis, no study on brolucizumab was included. There is paucity of data in literature on the use of brolucizumab in the treatment of RAP lesions. Only a retrospective case series on 12 eyes showed a good short-term efficacy of brolucizumab in reducing the size of type 3 neovascular membranes. (Gigon et al., 2022).

The present study presents some limitations. First, significant heterogeneity was found among included studies. This could limit the strength of our findings. A possible reason for high heterogeneity could be the variability in clinical characteristics and treatment protocols between included studies. However, all studies based RAP diagnosis on fluorescein and indocyanine green angiography and most included studies adopted a protocol treatment based on 3 monthly injections followed by a pro re nata regimen. Second, only two randomized clinical trials were included in this systematic review, of which only one provided data included in our pooled analyses. Furthermore, quantitative analyses were carried out from tabulated data extracted from each study because no individual data was available. However, confidence intervals yielded by meta-analysis studies are more powered and more accurate compared with individual studies. (Fallico et al., 2020c; Fallico et al., 2021). Finally, we could conduct meta-analyses only on data from a 12-month follow-up because data at a longer follow-up were provided by few studies. Pooled analyses of data with a long-term follow-up could have offered further insights in this issue and help to understand whether a combined approach could maintain functional and anatomical advantages in a such long-term. In conclusion, our analyses revealed, even if with a limited evidence, that the use of a combined approach with intravitreal anti-VEGF therapy and PDT could provide better functional and anatomical outcomes in RAP treatment. Such a combined approach seems to reduce the number of anti-VEGF injections, which could be a relevant advantage for both healthcare provider and patients. Further large randomized trials are needed to corroborate these findings and to investigate the role of new anti-VEGF drugs in this scenario.

## Data Availability

The original contributions presented in the study are included in the article/Supplementary Material, further inquiries can be directed to the corresponding author.

## References

[B1] Arias L. Cervera E. Vilimelis J. C. Escobar J. J. Escobar A. G. Zapata M. Á. (2020). Efficacy and safety of A treat-and-extend regimen with aflibercept in treatment-naive patients with type 3 neovascularization: A 52-week, single-arm, multicenter trial. Retina 40 (7), 1234–1244. 10.1097/IAE.0000000000002582 31259813

[B2] Arias L. Gómez-Ulla F. Ruiz-Moreno J. M. (2016). Ranibizumab in monotherapy and combined with photodynamic therapy for retinal angiomatous proliferation. Clin. Ophthalmol. 10, 861–869. 10.2147/OPTH.S106092 27274190 PMC4876105

[B4] Atmani K. Voigt M. Le Tien V. Querques G. Coscas G. Soubrane G. (2010). Ranibizumab for retinal angiomatous proliferation in age-related macular degeneration. Eye (Lond). 24 (7), 1193–1198. 10.1038/EYE.2010.9 20150927

[B5] Boscia F. Parodi M. B. Furino C. Reibaldi M. Sborgia C. (2006). Photodynamic therapy with verteporfin for retinal angiomatous proliferation. Graefes Arch. Clin. Exp. Ophthalmol. 244 (10), 1224–1232. 10.1007/S00417-005-0205-2 16525824

[B3] Bressler N. M. (2001). Treatment of age-related macular degeneration with photodynamic Therapy (TAP) study group. Photodynamic therapy of subfoveal choroidal neovascularization in age-related macular degeneration with verteporfin: two-year results of 2 randomized clinical trials-tap report 2. Arch. Ophthalmol. 119 (2), 198–207. 11176980

[B6] Browning A. C. O’brien J. M. Vieira R. V. Gupta R. Nenova K. (2019). Intravitreal aflibercept for retinal angiomatous proliferation: Results of a prospective case series at 96 weeks. Ophthalmologica 242 (4), 239–246. 10.1159/000500203 31163436

[B7] Cho H. J. Lee T. G. Han S. Y. Kim H. S. Kim J. H. Han J. I. (2016). Long-term visual outcome and prognostic factors of Intravitreal anti-vascular endothelial growth factor treatment for retinal angiomatous proliferation. Graefes Arch. Clin. Exp. Ophthalmol. 254 (1), 23–30. 10.1007/S00417-015-2993-3 25825231

[B8] Costagliola C. Romano M. R. dell’Omo R. Cipollone U. Polisena P. (2007). Intravitreal bevacizumab for the treatment of retinal angiomatous proliferation. Am. J. Ophthalmol. 144 (3), 449–451. 10.1016/J.AJO.2007.05.025 17765426

[B9] Engelbert M. Zweifel S. A. Freund K. B. (2009). Treat and extend dosing of intravitreal antivascular endothelial growth factor therapy for type 3 neovascularization/retinal angiomatous proliferation. Retina 29 (10), 1424–1431. 10.1097/IAE.0B013E3181BFBD46 19898180

[B10] Ernest J. Manethova K. Kolar P. Sobisek L. Sacconi R. Querques G. (2020). One-year results of fixed aflibercept treatment regime in type 3 neovascularization. Ophthalmologica 243 (1), 58–65. 10.1159/000499719 31121590

[B11] Fallico M. Chronopoulos A. Schutz J. S. Reibaldi M. (2020). Treatment of radiation maculopathy and radiation-induced macular edema: A systematic review. Surv. Ophthalmol. 66, 441–460. 10.1016/j.survophthal.2020.08.007 32918934

[B12] Fallico M. Lotery A. J. Longo A. Avitabile T. Bonfiglio V. Russo A. (2020). Treat and extend versus fixed regimen in neovascular age related macular degeneration: A systematic review and meta-analysis. Eur. J. Ophthalmol. 31, 2496–2504. 10.1177/1120672120964699 33118382

[B13] Fallico M. Maugeri A. Lotery A. Longo A. Bonfiglio V. Russo A. (2020). Intravitreal anti-vascular endothelial growth factors, panretinal photocoagulation and combined treatment for proliferative diabetic retinopathy: A systematic review and network meta-analysis. Acta Ophthalmol. 99, e795. 10.1111/aos.14681 33326183

[B14] Fallico M. Maugeri A. Lotery A. Longo A. Bonfiglio V. Russo A. (2021). Fluocinolone acetonide vitreous insert for chronic diabetic macular oedema: A systematic review with meta-analysis of real-world experience. Sci. Rep. 11 (1), 4800. 10.1038/s41598-021-84362-y 33637841 PMC7910468

[B15] Freund K. B. Van Ho I. Barbazetto I. A. Koizumi H. Laud K. Ferrara D. (2008). Type 3 neovascularization: The expanded spectrum of retinal angiomatous proliferation. Retina 28 (2), 201–211. 10.1097/IAE.0B013E3181669504 18301024

[B16] Gharbiya M. Parisi F. Cruciani F. Bozzoni-Pantaleoni F. Pranno F. Abdolrahimzadeh S. (2014). Intravitreal anti-vascular endothelial growth factor for retinal angiomatous proliferation in treatment-naive eyes: Long-term functional and anatomical results using a modified PrONTO-style regimen. Retina 34 (2), 298–305. 10.1097/IAE.0B013E3182979E62 23807188

[B17] Ghazi N. G. Conway B. P. (2005). Retinal angiomatous proliferation with a cilioretinal artery anastomosis: An unusual presentation. Graefes Arch. Clin. Exp. Ophthalmol. 243 (5), 493–496. 10.1007/S00417-004-1034-4 15931547

[B18] Gigon A. Vadalà M. Bonfiglio V. M. E. Reibaldi M. Eandi C. M. (2022). Early OCTA changes of type 3 macular neovascularization following brolucizumab intravitreal injections. Med. Kaunas. 58 (9), 1180. 10.3390/MEDICINA58091180 PMC9506440 36143855

[B19] Gupta B. Jyothi S. Sivaprasad S. (2010). Current treatment options for retinal angiomatous proliferans (RAP). Br. J. Ophthalmol. 94 (6), 672–677. 10.1136/BJO.2009.166975 19897475

[B20] Haj Najeeb B. Deak G. G. Schmidt-Erfurth U. M. Gerendas B. S. (2021). RAP study, report 1: Novel subtype of macular neovascularisation type III, cilioretinal MNV3. Br. J. Ophthalmol. 105 (1), 113–117. 10.1136/BJOPHTHALMOL-2019-315311 32161004

[B21] Hata M. Yamashiro K. Oishi A. Ooto S. Tamura H. Miyata M. (2017). Retinal pigment epithelial atrophy after anti-vascular endothelial growth factor injections for retinal angiomatous proliferation. Retina 37 (11), 2069–2077. 10.1097/IAE.0000000000001457 28085772

[B22] Hemeida T. S. Keane P. A. Dustin L. Sadda S. R. Fawzi A. A. (2010). Long-term visual and anatomical outcomes following anti-VEGF monotherapy for retinal angiomatous proliferation. Br. J. Ophthalmol. 94 (6), 701–705. 10.1136/BJO.2009.167627 19854733 PMC2878743

[B23] Higgins J. P. T. 2022 Cochrane handbook for systematic reviews of interventions New Jersey: John Wiley.

[B24] Inoue M. Arakawa A. Yamane S. Kadonosono K. (2014). Long-term results of intravitreal ranibizumab for the treatment of retinal angiomatous proliferation and utility of an advanced RPE analysis performed using spectral-domain optical coherence tomography. Br. J. Ophthalmol. 98 (7), 956–960. 10.1136/BJOPHTHALMOL-2013-304251 24599418

[B25] Invernizzi A. Teo K. Nguyen V. Daniell M. Squirrell D. Barthelmes D. (2019). Type 3 neovascularisation (retinal angiomatous proliferation) treated with antivascular endothelial growth factor: Real-world outcomes at 24 months. Br. J. Ophthalmol. 103 (9), 1337–1341. 10.1136/BJOPHTHALMOL-2018-312944 30504490

[B26] Kim J. H. Chang Y. S. Kim J. W. Kim C. G. Lee D. W. (2019). Abrupt visual loss during anti-vascular endothelial growth factor treatment for type 3 neovascularization. Int. J. Ophthalmol. 12 (3), 480–487. 10.18240/IJO.2019.03.20 30918819 PMC6423401

[B27] Kim J. H. Chang Y. S. Kim J. W. Kim C. G. Lee D. W. Cho S. Y. (2018). Difference in treatment outcomes according to optical coherence tomography-based stages in type 3 neovascularization (retinal angiomatous proliferation). Retina 38 (12), 2356–2362. 10.1097/IAE.0000000000001876 29019795

[B28] Kim J. H. Chang Y. S. Kim J. W. Kim C. G. Lee D. W. (2017). Recurrence in patients with type 3 neovascularization (retinal angiomatous proliferation) after intravitreal ranibizumab. Retina 37 (8), 1508–1515. 10.1097/IAE.0000000000001383 27787444

[B29] Kim J. H. Kim J. W. Kim C. G. Lee D. W. (2020). Long-term treatment outcomes in type 3 neovascularization: Focus on the difference in outcomes between geographic atrophy and fibrotic scarring. J. Clin. Med. 9 (4), 1145. 10.3390/JCM9041145 32316276 PMC7230588

[B30] Kim J. H. (2020). Results of switching from pro Re nata to treat-and-extend regimen in treatment of patients with type 3 neovascularization. Semin. Ophthalmol. 35 (1), 33–40. 10.1080/08820538.2019.1701045 31814497

[B31] Kim J. M. Kim J. H. Chang Y. S. Kim J. W. Kim C. G. Lee D. W. (2017). Treatment of bilateral retinal angiomatous proliferation with anti-vascular endothelial growth factor: 12-Month outcome. Korean J. Ophthalmol. 31 (3), 240–248. 10.3341/KJO.2016.0026 28471100 PMC5469927

[B32] Lee M. Y. Kim K. S. Lee W. K. (2011). Combination therapy of ranibizumab and photodynamic therapy for retinal angiomatous proliferation with serous pigment epithelial detachment in Korean patients: Twelve-month results. Retina 31 (1), 65–73. 10.1097/IAE.0B013E3181E586E3 21187732

[B33] Liberati A. Altman D. G. Tetzlaff J. Mulrow C. Gøtzsche P. C. Ioannidis J. P. A. (2009). The PRISMA statement for reporting systematic reviews and meta-analyses of studies that evaluate healthcare interventions: Explanation and elaboration. BMJ 339, b2700. 10.1136/bmj.b2700 19622552 PMC2714672

[B34] Lim T. H. Lai T. Y. Y. Takahashi K. Wong T. Y. Chen L. J. Ruamviboonsuk P. (2020). Comparison of ranibizumab with or without verteporfin photodynamic therapy for polypoidal choroidal vasculopathy: The EVEREST II randomized clinical trial. JAMA Ophthalmol. 138 (9), 935–942. 10.1001/JAMAOPHTHALMOL.2020.2443 32672800 PMC7366282

[B35] Malamos P. Tservakis I. Kanakis M. Koutsiouki C. Kiskira E. Mylonas G. (2018). Long-term results of combination treatment with single-dose ranibizumab plus photodynamic therapy for retinal angiomatous proliferation. Ophthalmologica 240 (4), 213–221. 10.1159/000487610 29768269

[B36] Maruyama-Inoue M. Sato S. Yamane S. Kadonosono K. (2019). Predictive factors and long-term visual outcomes after anti-vascular endothelial growth factor treatment of retinal angiomatous proliferation. Clin. Ophthalmol. 13, 1981–1989. 10.2147/OPTH.S224319 31631966 PMC6790115

[B37] Matsumoto H. Sato T. Morimoto M. Mukai R. Takahashi M. Hiroe T. (2016). Treat-and-extend regimen with aflibercept for retinal angiomatous proliferation. Retina 36 (12), 2282–2289. 10.1097/IAE.0000000000001104 27336229

[B38] Montero J. A. Fernandez M. I. Gomez-Ulla F. Ruiz-Moreno J. M. (2009). Efficacy of intravitreal bevacizumab to treat retinal angiomatous proliferation stage II and III. Eur. J. Ophthalmol. 19 (3), 448–451. 10.1177/112067210901900320 19396793

[B39] Nakano S. Honda S. Oh H. Kita M. Negi A. (2012). Effect of photodynamic therapy (PDT), posterior subtenon injection of triamcinolone acetonide with PDT, and intravitreal injection of ranibizumab with PDT for retinal angiomatous proliferation. Clin. Ophthalmol. 6 (1), 277–282. 10.2147/OPTH.S29718 22375096 PMC3287414

[B40] Park Y. G. Roh Y. J. (2015). One year results of intravitreal ranibizumab monotherapy for retinal angiomatous proliferation: A comparative analysis based on disease stages. BMC Ophthalmol. 15 (1), 182. 10.1186/S12886-015-0172-2 26691185 PMC4685625

[B41] Parodi M. B. Iacono P. Menchini F. Sheth S. Polini G. Pittino R. (2013). Intravitreal bevacizumab versus ranibizumab for the treatment of retinal angiomatous proliferation. Acta Ophthalmol. 91 (3), 267–273. 10.1111/J.1755-3768.2011.02265.X 21951313

[B42] Reche-Frutos J. Calvo-Gonzalez C. Pérez-Trigo S. Fernandez-Perez C. Donate-Lopez J. Garcia-Feijoo J. (2011). Ranibizumab in retinal angiomatous proliferation (RAP): Influence of RAP stage on visual outcome. Eur. J. Ophthalmol. 21 (6), 783–788. 10.5301/EJO.2011.6526 21484755

[B43] Reibaldi M. Cardascia N. Longo A. Furino C. Avitabile T. Faro S. (2010). Standard-fluence versus low-fluence photodynamic therapy in chronic central serous chorioretinopathy: A nonrandomized clinical trial. Am. J. Ophthalmol. 149 (2), 307–315. 10.1016/J.AJO.2009.08.026 19896635

[B44] Reibaldi M. Fallico M. Avitabile T. Bonfiglio V. Russo A. Castellino N. (2020). Risk of death associated with intravitreal anti-vascular endothelial growth factor therapy: A systematic review and meta-analysis. JAMA Ophthalmol. 138 (1), 50–57. 10.1001/jamaophthalmol.2019.4636 31750861 PMC6902107

[B45] Reibaldi M. Fallico M. Avitabile T. Marolo P. Parisi G. Cennamo G. (2022). Frequency of intravitreal anti-VEGF injections and risk of death: A systematic review with meta-analysis. Ophthalmol. Retin 6 (5), 369–376. 10.1016/j.oret.2021.12.019 34974177

[B46] Rouvas A. A. Chatziralli I. P. Theodossiadis P. G. Moschos M. M. Kotsolis A. I. Ladas I. D. (2012). Long-term results of intravitreal ranibizumab, intravitreal ranibizumab with photodynamic therapy, and intravitreal triamcinolone with photodynamic therapy for the treatment of retinal angiomatous proliferation. Retina 32 (6), 1181–1189. 10.1097/IAE.0B013E318235D8CE 22466469

[B47] Saito M. Iida T. Kano M. (2012). Combined intravitreal ranibizumab and photodynamic therapy for retinal angiomatous proliferation. Am. J. Ophthalmol. 153 (3), 504–514. 10.1016/J.AJO.2011.08.038 22078902

[B48] Saito M. Iida T. Kano M. Itagaki K. (2016). Two-year results of combined intravitreal ranibizumab and photodynamic therapy for retinal angiomatous proliferation. Jpn. J. Ophthalmol. 60 (1), 42–50. 10.1007/S10384-015-0417-X 26498642

[B49] Saito M. Iida T. Kano M. (2013). Two-year results of combined intravitreal anti-VEGF agents and photodynamic therapy for retinal angiomatous proliferation. Jpn. J. Ophthalmol. 57 (2), 211–220. 10.1007/S10384-012-0215-7 23208024

[B50] Saito M. Shiragami C. Shiraga F. Kano M. Iida T. (2010). Comparison of intravitreal triamcinolone acetonide with photodynamic therapy and intravitreal bevacizumab with photodynamic therapy for retinal angiomatous proliferation. Am. J. Ophthalmol. 149 (3), 472–481. 10.1016/J.AJO.2009.09.016 20053392

[B51] Seidel G. Werner C. Weger M. Steinbrugger I. Haas A. (2013). Combination treatment of photodynamic therapy with verteporfin and intravitreal ranibizumab in patients with retinal angiomatous proliferation. Acta Ophthalmol. 91 (6), e482–e485. 10.1111/AOS.12111 23786546

[B52] Shin J. Y. Yu H. G. (2014). Optical coherence tomography-based ranibizumab monotherapy for retinal angiomatous proliferation in Korean patients. Retina 34 (12), 2359–2366. 10.1097/IAE.0000000000000225 25011025

[B53] Slim K. Nini E. Forestier D. Kwiatkowski F. Panis Y. Chipponi J. (2003). Methodological index for non-randomized studies (Minors): Development and validation of a new instrument. ANZ J. Surg. 73 (9), 712–716. 10.1046/j.1445-2197.2003.02748.x 12956787

[B54] Tsai A. S. H. Cheung N. Gan A. T. L. Jaffe G. J. Sivaprasad S. Wong T. Y. (2017). Retinal angiomatous proliferation. Surv. Ophthalmol. 62 (4), 462–492. 10.1016/J.SURVOPHTHAL.2017.01.008 28189495

[B55] Viola F. Massacesi A. Orzalesi N. Ratiglia R. Staurenghi G. (2009). Retinal angiomatous proliferation: Natural history and progression of visual loss. Retina 29 (6), 732–739. 10.1097/IAE.0B013E3181A395CB 19516115

[B56] Yannuzzi L. A. Freund K. B. Takahashi B. S. (2008). Review of retinal angiomatous proliferation or type 3 neovascularization. Retina 28 (3), 375–384. 10.1097/IAE.0B013E3181619C55 18327130

[B57] Yannuzzi L. A. Negrão S. Iida T. Carvalho C. Rodriguez-Coleman H. Slakter J. (2001). Retinal angiomatous proliferation in age-related macular degeneration. Retina 21 (5), 416–434. 10.1097/00006982-200110000-00003 11642370

