# Supplementary figures and images for: Anti-vascular endothelial growth factor monotherapy or combined with verteporfin photodynamic therapy for retinal angiomatous proliferation: a systematic review with meta-analysis
Source: Front Pharmacol. 2023 Jun 12;14:1141077. doi: 10.3389/fphar.2023.1141077 (PMC10291099; doi:10.3389/fphar.2023.1141077)

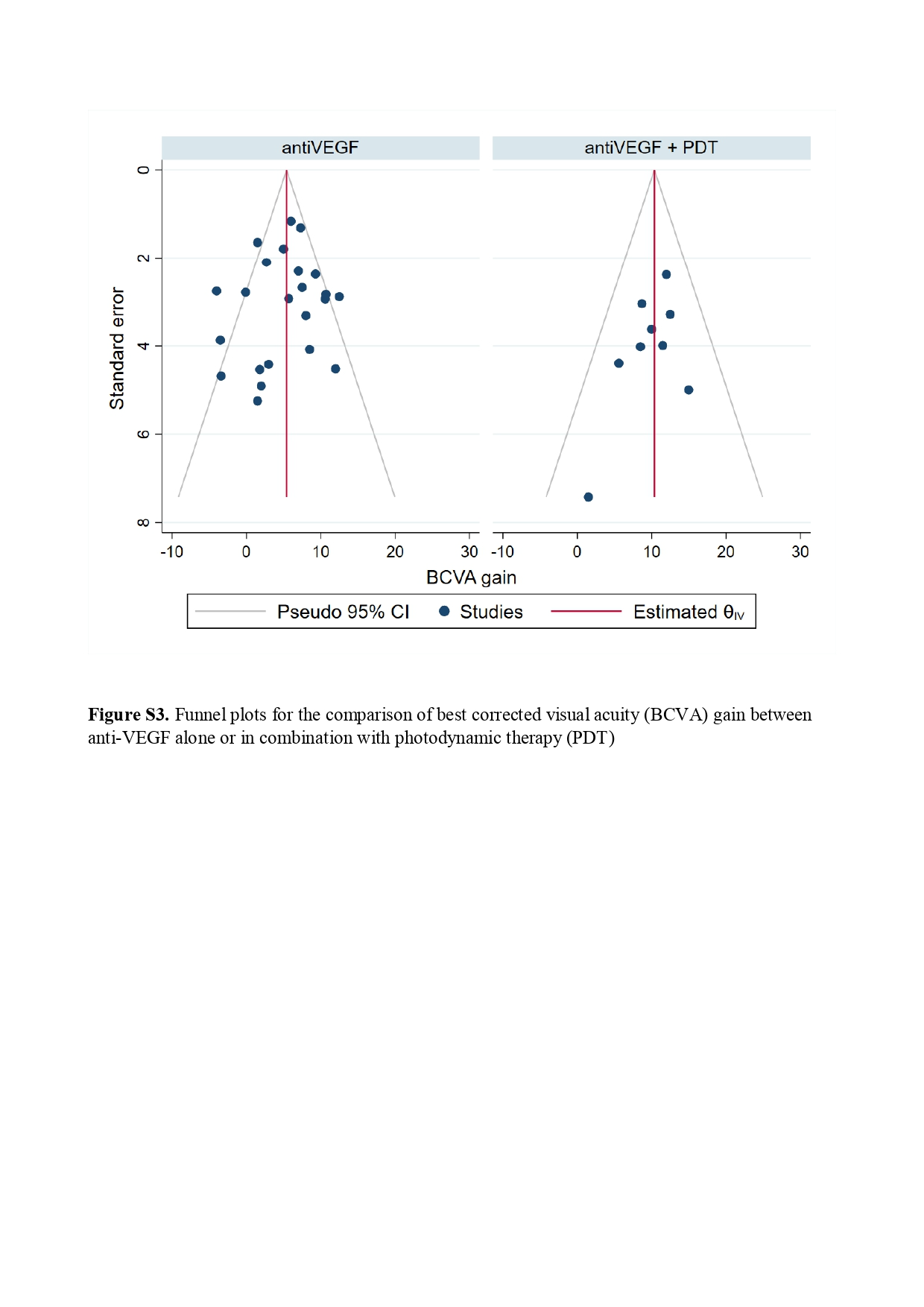

Supplement: Supplementary file 1 [file Image3.TIFF]

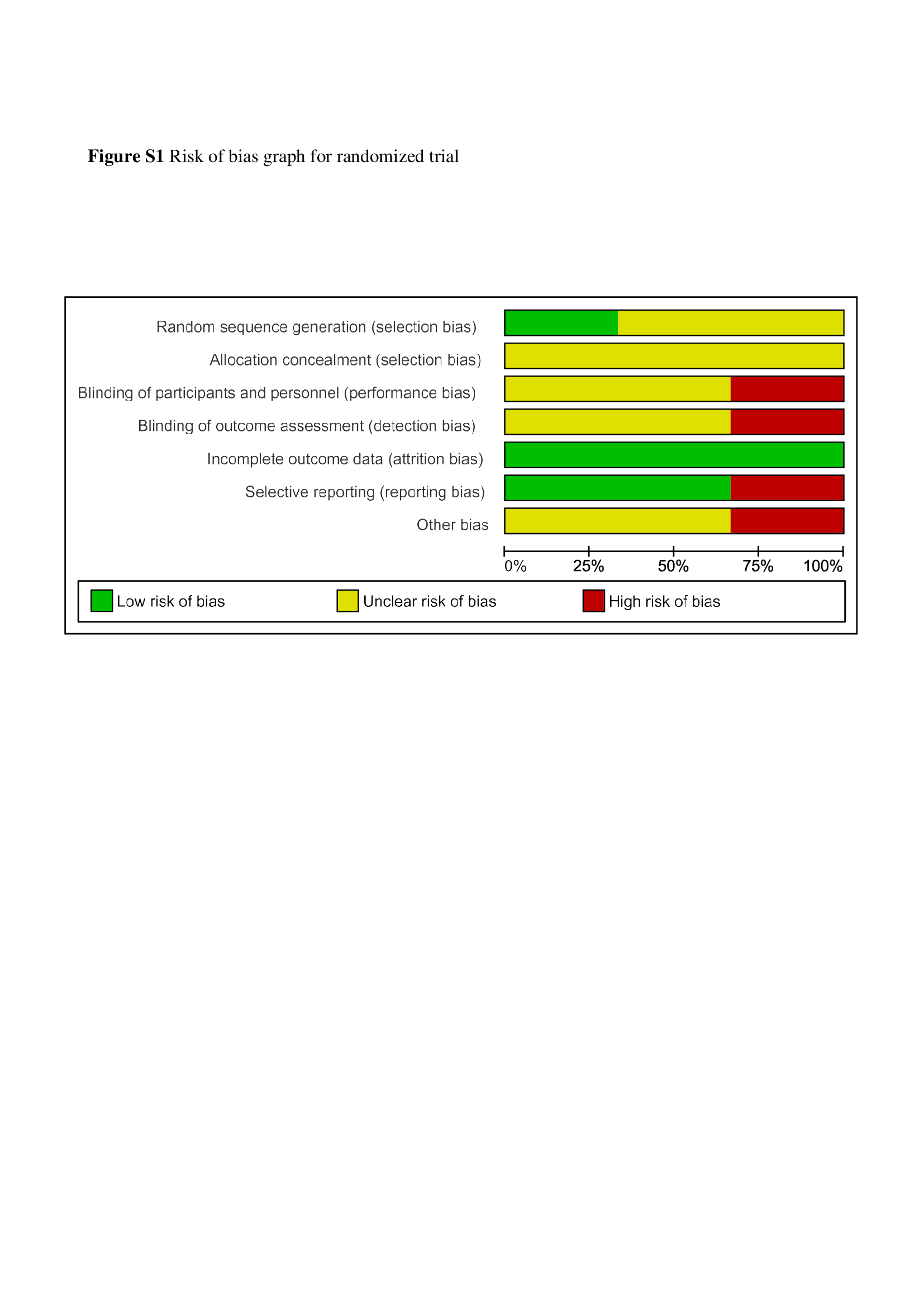

Supplement: Supplementary file 2 [file Image1.TIFF]

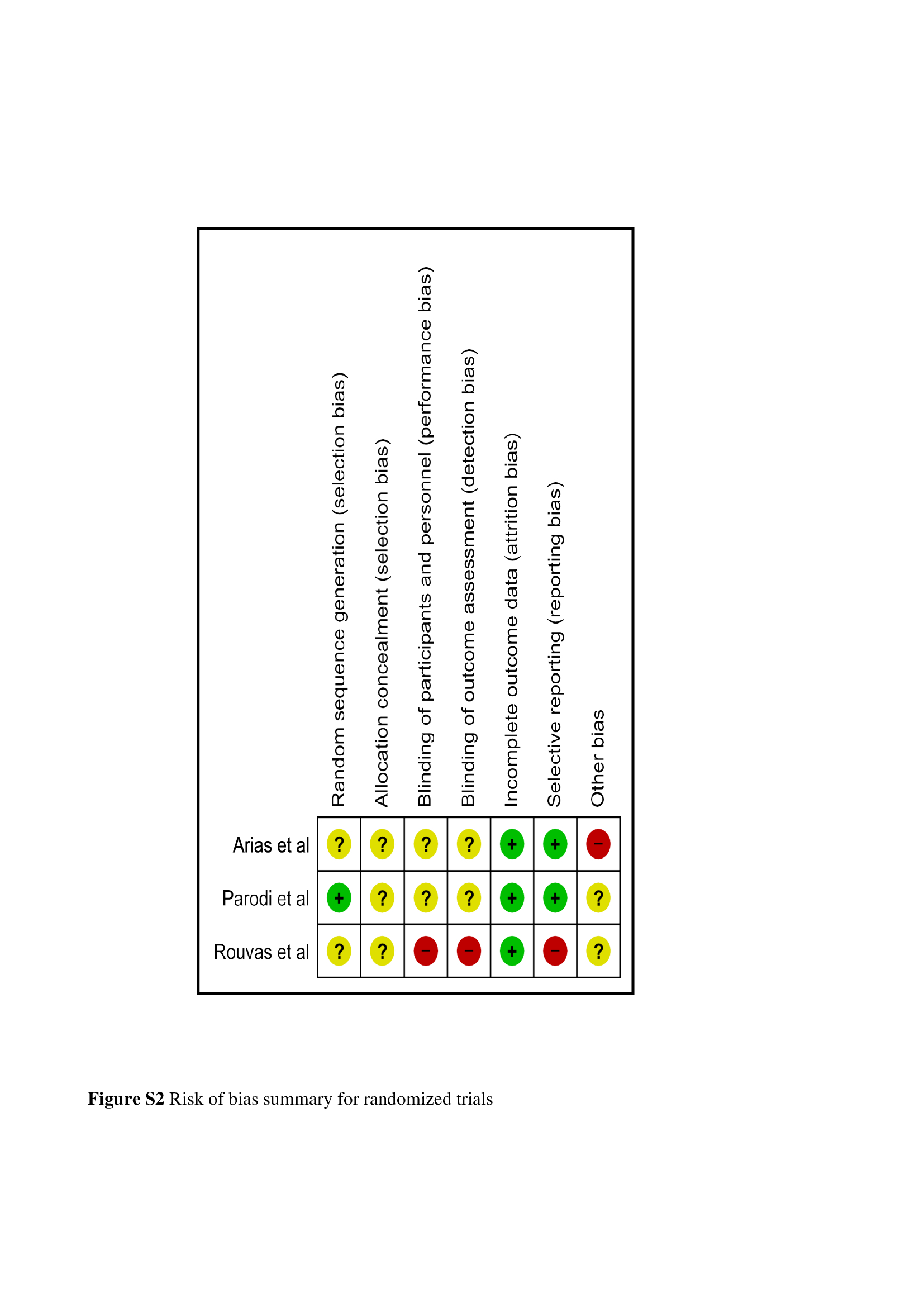

Supplement: Supplementary file 4 [file Image2.TIFF]

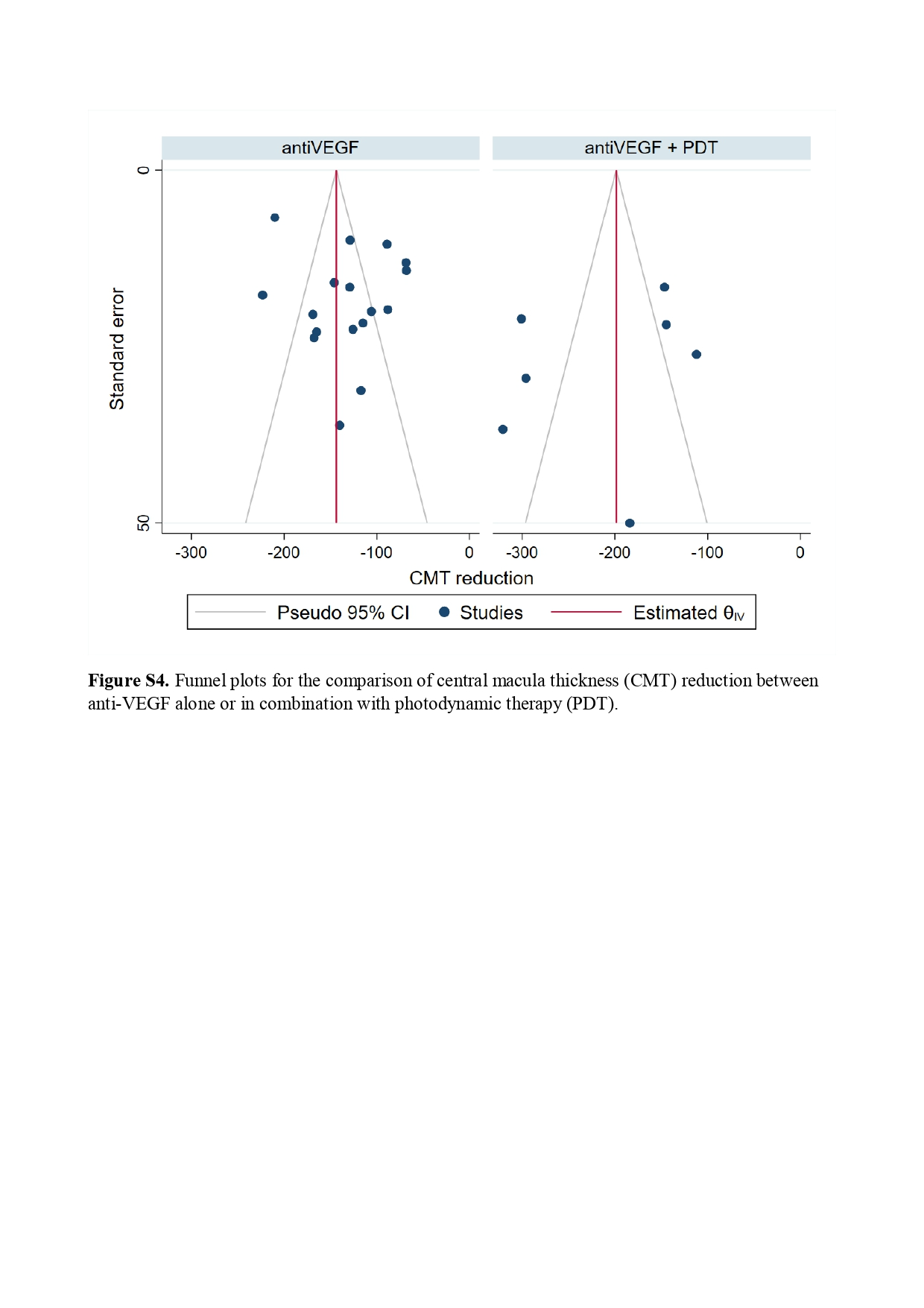

Supplement: Supplementary file 5 [file Image4.TIFF]
