# Supplementary material for: Anti-vascular endothelial growth factor monotherapy or combined with verteporfin photodynamic therapy for retinal angiomatous proliferation: a systematic review with meta-analysis
Source: Front Pharmacol. 2023 Jun 12;14:1141077. doi: 10.3389/fphar.2023.1141077 (PMC10291099; doi:10.3389/fphar.2023.1141077)
Supplement: Supplementary file 3 [file DataSheet1.pdf]

List of elements included in the Supplement

Table S1 Methodological item for non-randomized studies (MINORS) score for non-randomized studies

Figure S1 Risk of bias graph for randomized trials

Figure S2 Risk of bias summary for randomized trials

Figure S3 Funnel plot showing best corrected visual acuity (BCVA) gain after one year of treatment with anti-VEGF alone or in combination with photodynamic therapy (PDT

Figure S4 Funnel plot showing central macular thickness (CMT) reduction after one year of treatment with anti-VEGF alone or in combination with photodynamic therapy (PDT)

Table S1. Methodological item for non-randomized studies (MINORS) score for non-randomized studies

Author, year	Clearly stated aim	Consecutive patients	Prospective data collection	Endpoints appropriate to the aim of the study	Unbiased assessment of the study endpoint	Follow-up period appropriate to the aim of the study	Loss to follow up less than 5%	Prospective calculation of the study size	An adequate control group	Contemporary groups	Baseline equivalence of groups	Adequate statistical analyses	Total
Montero et al 2009	2	0	0	1	2	2	2	0	NA non c'è	NA	NA	2	11
Atmani et al 2010	2	2	2	2	2	2	2	0	NA	NA	NA	2	16
Parodi et al 2013	2	2	2	2	2	2	2	0	NA	NA	NA	2	16
Reche-Frutos et al 2011	2	2	2	2	2	2	2	0	NA	NA	NA	2	16
Shin et al 2014	2	NA	2	2	2	2	2	0	NA	NA	NA	2	14
Gharbiya et al 2014	2	2	2	1	2	2	1	0	0	0	NA	2	14
Park et al 2015	2	NA	0	2	2	2	NA	0	NA	2	NA	2	12
Inoue et al 2014	2	2	0	2	2	2	0	0	NA	NA	NA	2	12
Cho et al 2015	2	NA	2	2	2	2	1	0	NA	2	NA	2	15

Author, year	Clearly stated aim	Consecutive patients	Prospective data collection	Endpoints appropriate to the aim of the study	Unbiased assessment of the study endpoint	Follow-up period appropriate to the aim of the study	Loss to follow up less than 5%	Prospective calculation of the study size	An adequate control group	Contemporary groups	Baseline equivalence of groups	Adequate statistical analyses	Total
Rouvas et al 2012	2	NA	2	2	2	2	0	0	NA	2	NA	2	14
Arias et al 2016	2	NA	2	2	2	2	2	0	NA	2	NA	2	16
Ernest et al 2019	2	0	2	2	2	2	NA	0	NA	NA	NA	2	12
Maruyama-Inoue et al 2019	2	0	0	2	2	2	0	0	NA	2	NA	2	12
Browning et al 2019	1	2	2	2	2	2	1 >5%	0	NA	NA	NA	2	14
Invernizzi et al 2019	2	0	2	1	2	2	NA	0	2	2	NA	2	15
Arias et al 2020	2	NA	2	2	2	2	1	2	NA	NA	NA	2	15
Kim et al 2017	2	0	0	1	2	2	2	0	NA	2	1	2	14
Engelbert et al 2009	2	2	0	2	2	2	2	0	NA	0	0	2	14

Author, year	clearly stated aim	consecutive patients	Prospective data collection	Endpoints appropriate to the aim of the study	Unbiased assessment of the study endpoint	Follow-up period appropriate to the aim of the study	Loss to follow up less than 5%	Prospective calculation of the study size	An adequate control group	Contemporary groups	Baseline equivalence of groups	Adequate statistical analyses	Totale
Hata et al 2017	2	2	0	2	2	2	2	0	NA	2	1	2	17
Kim et al 2019	2	0	0	1	2	2	0	0	NA	1	1	2	11
Kim et al 2017 (2018)	2	0	0	2	2	2	1	0	NA	1	1	2	13
Kim et al 2017	2	0	0	2	2	2	0	0	NA	NA	NA	2	10
Kim et al 2020	2	0	0	1	2	2	0	0	NA	1	1	2	11
Kim et al 2020	2	NA	0	1	2	2	2	0	NA	2	1	2	14
Saito et al. 2010	2	0	0	2	2	2	2	0	NA	2	0	2	14
Saito et al 2012	2	2	0	2	2	2	0	0	NA	NA	NA	2	12
Saito et al 2013	2	2	0	2	2	2	2	0	NA	2	1	2	17

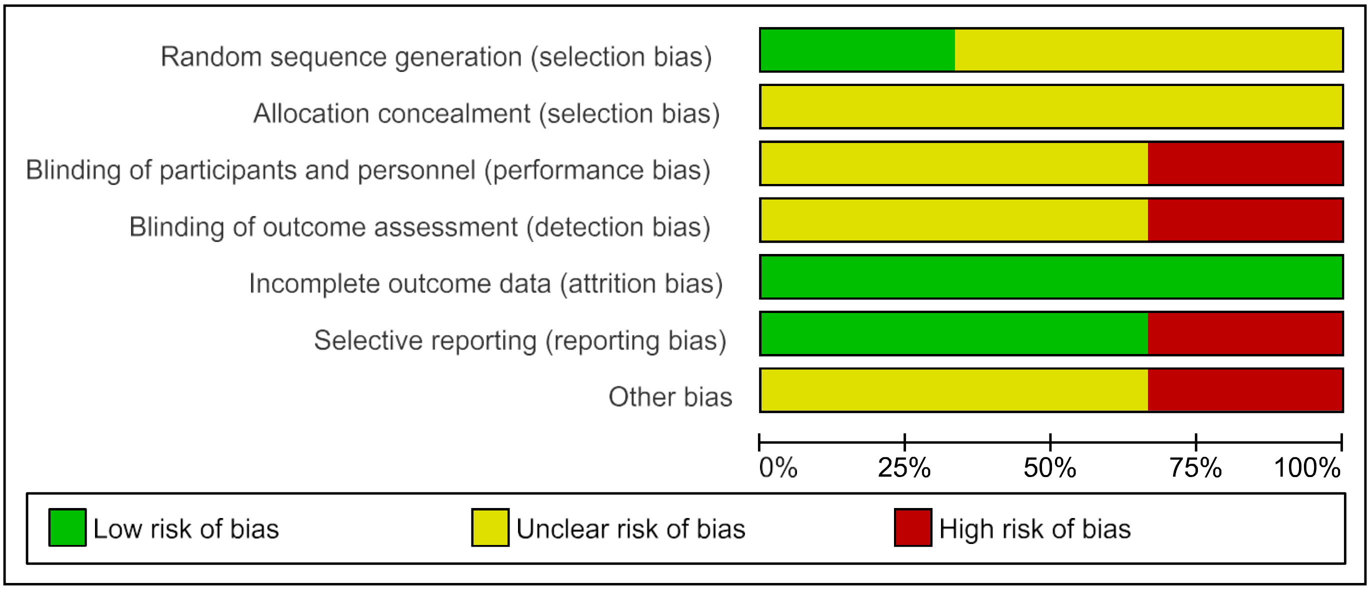

Figure S1 Risk of bias graph for randomized trial

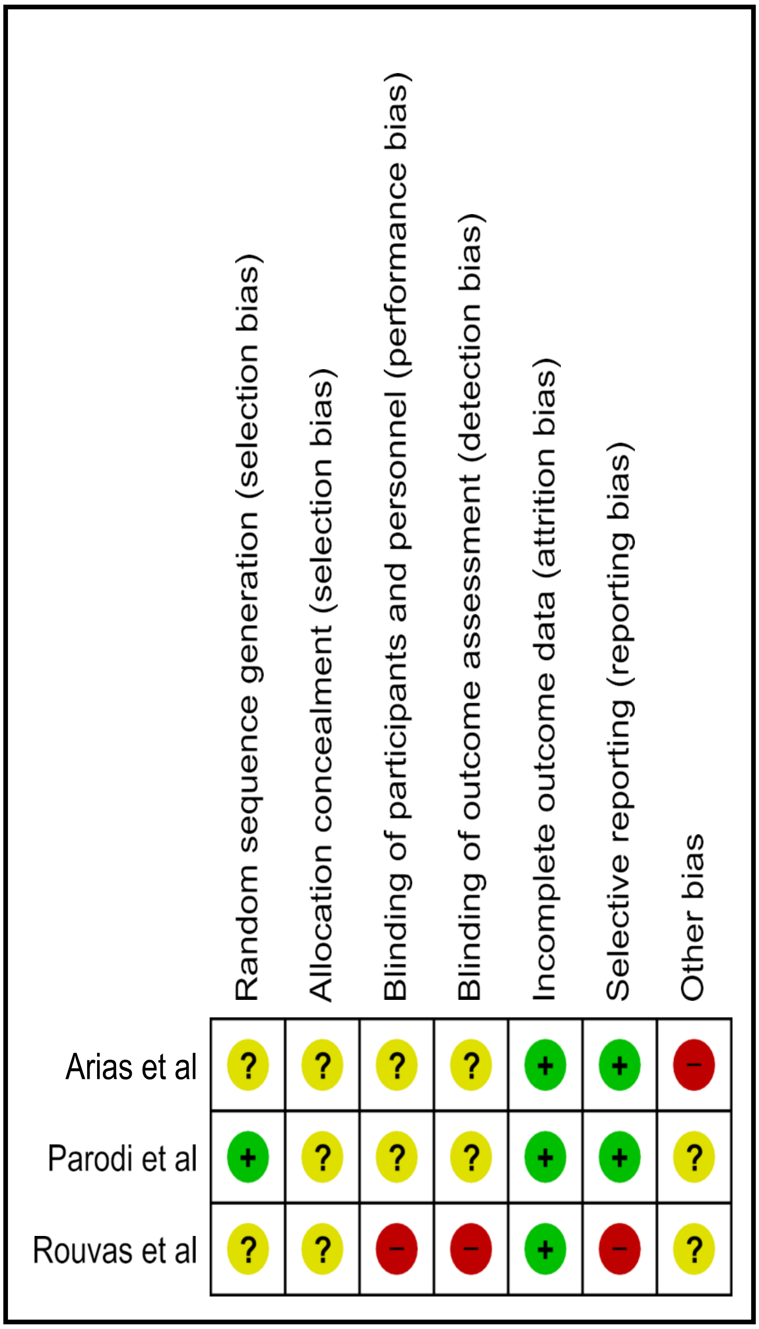

Figure S2 Risk of bias summary for randomized trials

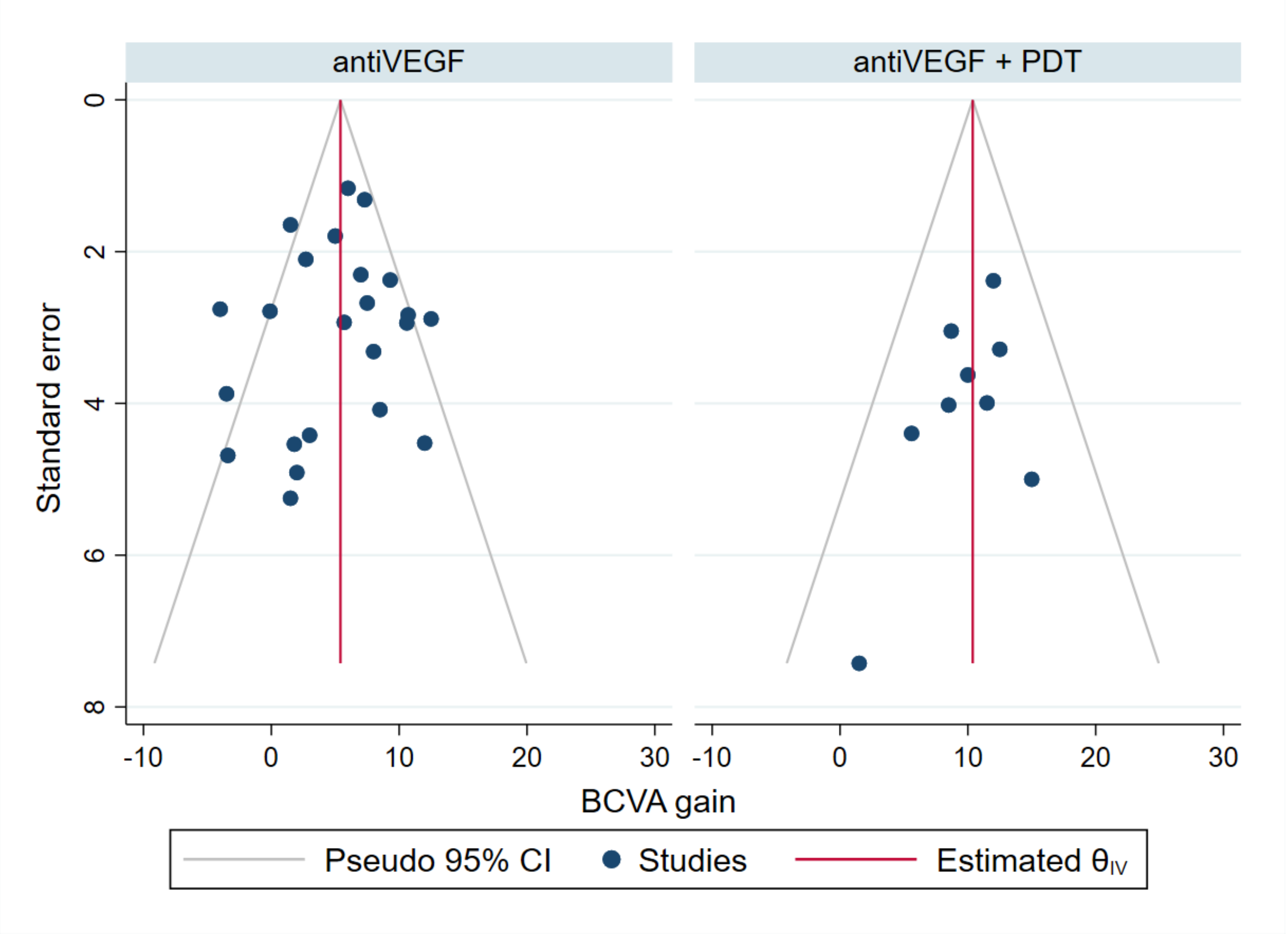

Figure S3. Funnel plots for the comparison of best corrected visual acuity (BCVA) gain between anti-VEGF alone or in combination with photodynamic therapy (PDT)

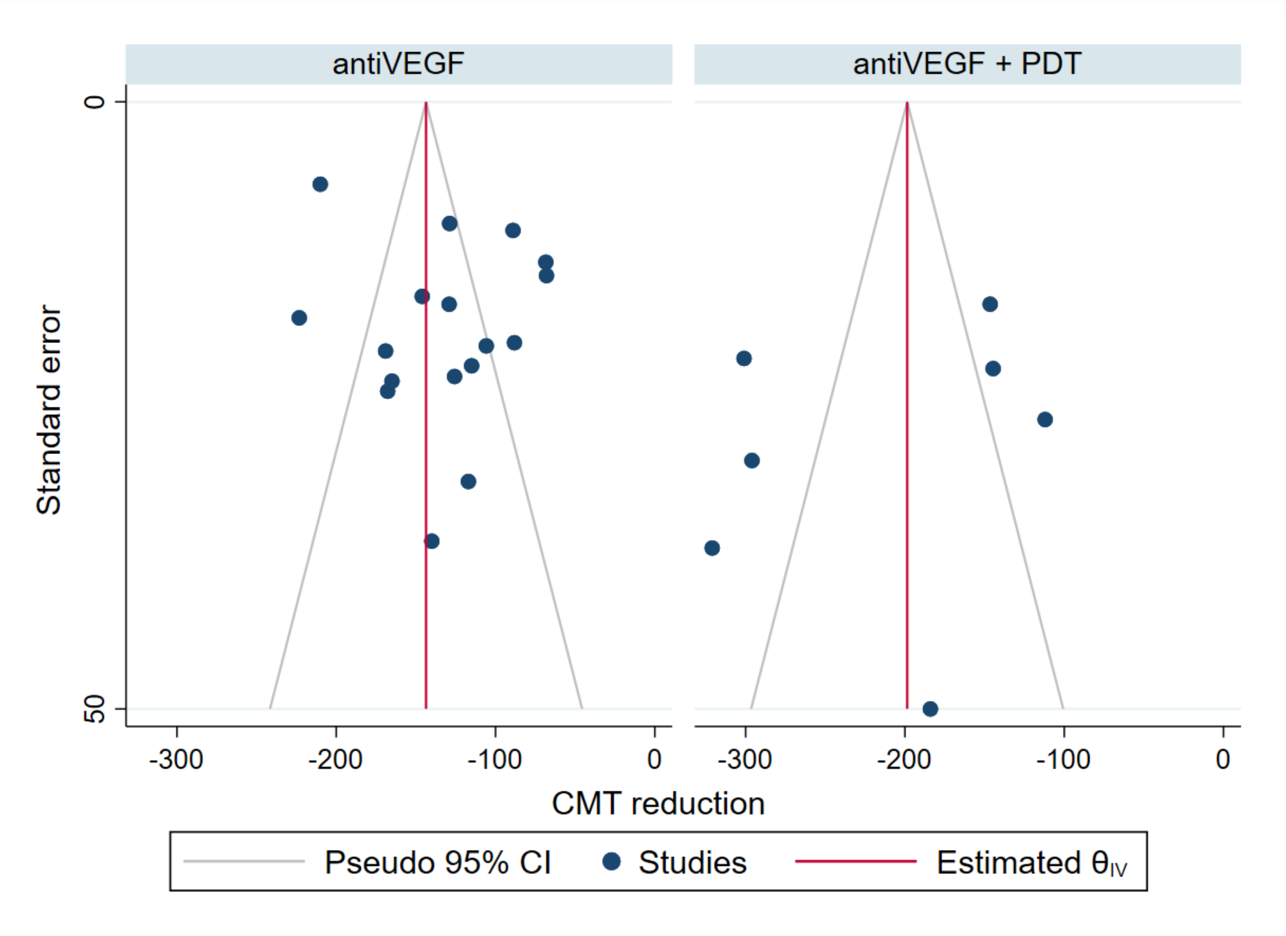

Figure S4. Funnel plots for the comparison of central macula thickness (CMT) reduction between anti-VEGF alone or in combination with photodynamic therapy (PDT).
